# Anti-inflammatory and Antimicrobial Properties of Ibuprofen Analogues Derived by Photoredox-Catalyzed C–N Scission of Tertiary Amines and Amidation

**DOI:** 10.1021/acsomega.5c11131

**Published:** 2026-02-25

**Authors:** Ozgur YILMAZ, Merve DOGAN, Derya YETKIN, Pinar KUCE CEVIK, Marion H. Emmert

**Affiliations:** a Department of Chemistry, Faculty of Sciences, 52983 Mersin University, Mersin 33343, Turkey; b Advanced Technology Research and Application Center, 52983 Mersin University, Mersin 33343, Turkey; c Department of Molecular Biology and Genetic, Faculty of Science and Arts, 52966 Harran University, Sanliurfa 63290, Turkey; d Discovery Chemistry, MRL, Merck & Co., Inc., 770 Sumneytown Pike, West Point, Pennsylvania 19486, United States

## Abstract

This manuscript describes the synthesis of ibuprofen-derived amides, and their biological evaluation with respect to anti-inflammatory and antimicrobial properties, including inhibition of biofilm formation. The compounds were synthesized using a previously developed photoredox-catalyzed protocol, which proceeds through C–N bond scission of the tertiary amine building blocks, followed by in situ amide coupling; thus, 16 derivatives of ibuprofen were prepared on 30 to 70 mg scales. Evaluation in multiple antimicrobial and biofilm-related biological assays revealed desired activities for many of the compounds. Compounds **9**–**11** and **14**–**16** are newly synthesized in the literature, compounds **1**–**8** have been previously reported by us, and compounds **12**–**13** are known in the literature. The broad-spectrum antimicrobial activities of all molecules were evaluated for the first time in these studies. An initial phenotypic screening under inflammatory stress was performed using an MTT-based LPS challenge model, which revealed that several derivatives (**2**, **4**, **7**, and **11**–**16**) preserved cell viability more effectively than ibuprofen. Based on these findings, selected compounds were further evaluated using direct biochemical assays, demonstrating that ibuprofen amide derivatives significantly suppressed LPS-induced production of the pro-inflammatory cytokines IL-1β, TNF-α, and IL-6 in RAW 264.7 macrophages. Assessment of antimicrobial activity against *Escherichia coli*, MRSA, and *Candida albicans* demonstrated notable inhibitory activities by compounds **15** and **16**. The same compounds also showed inhibition of biofilm growth. Collectively, these results demonstrate that photoredox-catalyzed modification of the ibuprofen scaffold enables rapid access to newly synthesized analogues combining confirmed anti-inflammatory activity with antimicrobial and antibiofilm properties.

## Graphical abstract

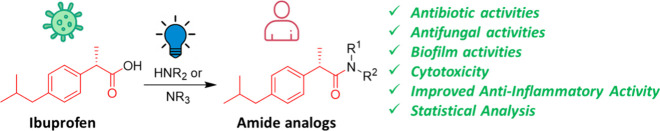

## Introduction

Inflammation plays a central role in the development of various acute and chronic diseases, including autoimmune disorders, cardiovascular conditions, and cancer.^1,2^ Nonsteroidal anti-inflammatory drugs (NSAIDs) are among the most commonly prescribed medications for managing pain, fever, and inflammation.^3,4^ This makes them indispensable in clinical practice. Ibuprofen (2-(4-isobutylphenyl)­propionic acid) is one of the most frequently used NSAIDs, valued for its efficacy, favorable safety profile, and over-the-counter availability.^5,6^

Beyond its well-established use in managing inflammation, pain, and fever, recent studies have examined the broader biological potential of ibuprofen and its derivatives. Many structural modifications of the ibuprofen scaffold have been used to produce close analogues, including the heterocycles or other functional groups such as amides,^7,8^ oxadiazoles,^9^ hydrazones,^10^ benzimidazole,^11^ oxazolone,^12^ furoxan,^13^ and Schiff bases.^5^ These modifications have been shown to enhance anti-inflammatory properties as well as introduce antimicrobial, antifungal, antioxidant and even anticancer activities.^14,15^ One reason to pursue analog synthesis in this space is to overcome the limitations of conventional NSAIDs, such as gastrointestinal toxicity.^3^ For example, stomach ulcer disorders^6,16^ have been traced back to effects of the acid moiety in ibuprofen.

Encouraged by this body of work, we targeted the synthesis of ibuprofen derivatives without acid functionalities. Specifically, we were interested in amide derivatives that can be obtained readily through amide bond formation. Amides are one of the most important functional groups in medicinal chemistry.^17–21^ Traditional methods for amide bond formation typically involve the activation of carboxylic acids using harsh reagents such as acid chlorides, anhydrides, or amide coupling agents.^22–24^ In recent years, visible-light-driven photoredox catalysis has provided a powerful alternative for the direct amidation of carboxylic acids with amines.^18,25–27^ Specifically, these protocols enable amide bond formation under milder reaction conditions, with high functional group tolerance.

This study demonstrates the synthesis of ibuprofen amide analogues through a photoredox-catalyzed protocol for amide formation from carboxylic acids and tertiary amines, proceeding via amine C–N bond scission.^18^ This approach rapidly provided access to new chemical entities. The evaluation of these analogues provides first insights into the biological activities of such derivatives. Based on previous literature examples, we reasoned that ibuprofen analogues without acid functionalities should be able to retain their anti-inflammatory activities, while limiting gastrointestinal toxicities that are typically attributed to the presence of an acid group in ibuprofen. Therefore, we targeted the synthesis of amide analogues of ibuprofen.

## Results and Discussion

### Chemistry

The synthesis of complex amides from tertiary amines and carboxylic acids has been previously reported by this author group, using an iridium-catalyzed, visible-light-promoted, aerobic protocol.^18^ CF_3_SO_2_Na serves to activate the carboxylic acid under the reaction conditions. We postulated that diverse amide derivatives of ibuprofen should be readily available with this method. Following the published procedure,^18^ we thus prepared compounds **1** to **9** from (*S*)-(+)-ibuprofen and tertiary amine building blocks (Scheme 1). As demonstrated previously on one example, the reaction also proceeds readily with secondary amines as building blocks, which allowed us to prepare derivatives **10** to **16**. The resulting set of ibuprofen analogues includes cyclic and acyclic functionalities on the amide moiety, including aromatic, heterocyclic, and alkyl groups. Amidation reactions generally proceeded with moderate to good yields ranging from 33 to 77% (with isolated yields ranging from 30 to 74%). Yields on the lower end of this spectrum were observed for compounds **9**, **14**, and **15**, likely reflecting the lowered nucleophilicity of the sterically bulky amine reactants. Overall, the method provides higher yields for tertiary amine substrates as compared to secondary amine substrates. With these compounds in hand, our next step focused on evaluating their biological activity.

**Scheme 1:**
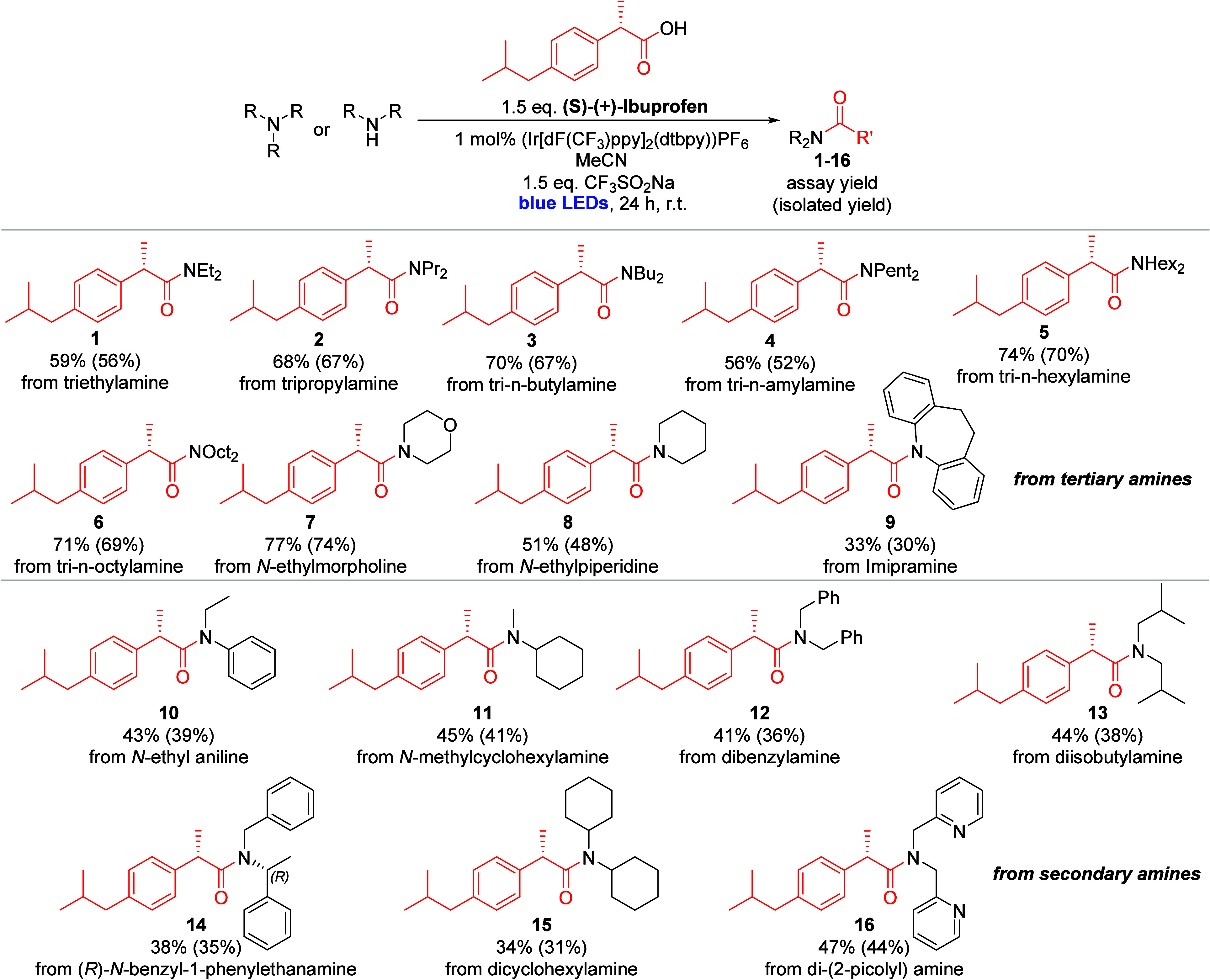
Synthetic Route and Obtained Yields for the Preparation of Ibuprofen-Derived Amides (**1**–**16**)

### Biology

#### Cytotoxicity, Anti-Inflammatory Potential, and Statistical Analysis

In order to assess the potential cytotoxicity of the prepared analogues, an MTT assay was performed, employing the RAW264.7 cell line (for details, see the Experimental Section). The results are shown in Figure 1 (for full numerical data, please see the Supporting Information (SI) file page S26/Table S1). All tested compounds, including ibuprofen, demonstrate a dose-dependent cytotoxic effect, which is significant at high concentrations (>500 μM). For some analogues (e.g., compound **14**), the onset of a cytotoxic effect occurs at even lower concentrations (∼70% cell viability at 100 μM). Several analogues show >50% cell survival at the highest tested concentration of 1000 μM (e.g., 81 ± 0.3% for **9**; 71 ± 1% for **10**), while cell viability dropped to 49 ± 2% at 1000 μM ibuprofen. Some of the new analogues showed even lower cell viability than ibuprofen at those high concentrations (e.g., 33 ± 4% for **4**). Generally, most values for the cytotoxicity of the new derivatives were measured to fall within a 3-fold range of the values obtained for ibuprofen itself (Figure 1). Based on these data, we chose 100 μM for further studies, as cell viability was 70% or greater for all compounds tested at this concentration.

**Figure 1:**
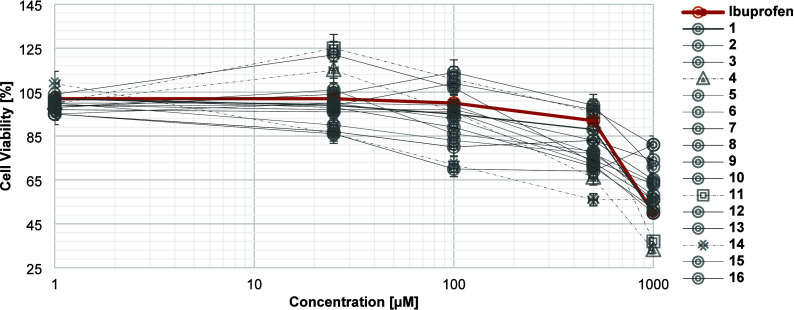
Cytotoxicity assessment for newly prepared compounds **1** to **16** and comparison with ibuprofen via MTT assay. Cell culture in high-glucose DMEM with 10% FBS and 1% penicillin–streptomycin. Assessment of cell viability after 48 h of treatment with compounds or ibuprofen at specified concentrations via MTT reagent treatment, 4 h incubation, formazan dissolution with DMSO, and absorbance measurement at 570 nm. Provided values are shown as the average of values obtained from four replicate measurements of the same assay plate.

First, we decided to test how the synthesized compounds compare to ibuprofen in terms of their effects on cell viability under inflammatory stress conditions. To this end, cells were first incubated with 100 μM solutions of the compounds for 2 h; then, an inflammatory response^28^ was induced by addition of LPS (lipopolysaccharide). After 22 h incubation, cell viability was measured again using the MTT assay. The resulting data (LPS+; light colored bars) and the comparison with cell viability values in the absence of LPS (LPS–; dark colored bars) are shown in Figure 2.

**Figure 2:**
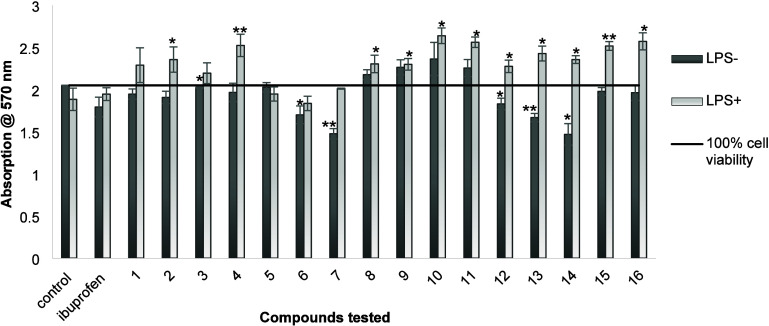
Comparison of MTT-based cell viability in RAW 264.7 macrophages in the absence (LPS−) and presence (LPS+) of LPS as immune activator, used to assess the effects of ibuprofen amide derivatives on cell viability under inflammatory stress conditions. Cells were pretreated with 100 μM of each compound (or left untreated in the control) for 2 h, followed by stimulation with 1 μg/mL LPS for 22 h. Data are presented as average values and standard deviations obtained from measurements of six replicate experiments. Statistical significance was determined at *P < 0.05 and **P < 0.001. Asterisks denote statistically significant differences of either the LPS– or LPS+ groups relative to the respective measurements in the control.

Overall, cell viability remained high in cells not subjected to LPS-induced stress (88–115% cell viability, with the control defined as 100% cell viability), with the exception of cultures treated with compound **7** (72 ± 3% cell viability) and **14** (72 ± 6% cell viability). Within this nonactivated group (shown as dark bars/LPS– in Figure 2), ibuprofen derivatives **8**, **9**, **10**, and **11** were associated with the highest levels of cell viability (106 ± 3, 111 ± 4, 115 ± 10, and 110 ± 5%, respectively). Following induction of an inflammatory response with LPS, cell viability in the control group decreased to 92 ± 6%, indicating the successful induction of an inflammatory-like condition. The group pretreated with ibuprofen shows a small decrease in cell viability in the absence of LPS (LPS–; 88 ± 6%) and high cell viability in the presence of LPS (LPS+; 95 ± 4%). This observation suggests a modest protective effect against LPS-induced inflammatory stress, even though the actual LPS– and LPS+ values are not statistically significantly different.

Excitingly, a number of ibuprofen derivatives demonstrated the ability to increase cell viability. Specifically, compounds **2**, **4**, **7**, and **11**–**16** all showed a statistically significant increase in cell viability, as defined by comparing the respective LPS+ and LPS– values. Interestingly, these findings indicate enhanced cytoprotective effects under LPS-induced inflammatory stress when compared to ibuprofen. Similar observations had been made previously for modified ibuprofen analogues.^3,10^ Overall, these results indicate that altering the acid functionality of ibuprofen can modulate cell viability and cell protective responses under inflammatory stress. This provides a rationale for a more detailed evaluation of the mechanism of its anti-inflammatory activities using more direct biochemical assays.

According to the analysis of cell viability under LPS-induced inflammatory stress (Figure 2), it is understood that activity varies depending on many parameters such as steric effect, polarity, and chain length. At first glance, most amide derivatives appear to be more effective than ibuprofen on cell viability under inflammatory stress conditions. Compounds **11**, **15**, and **16**, which have cyclic and moderately lipophilic substituted groups, showed improved cell viability under LPS-stimulated conditions. In contrast, compounds **12** and **14**, although they also contain steric substituent groups, displayed a less pronounced protective effect on cell viability compared to other molecules. This may be due to their limited cellular uptake, resulting from reduced solubility owing to their highly aromatic and less polar structures. In contrast, smaller alkyl amide derivatives with varying chain lengths (e.g., **1**, **3**, and **5**) showed more moderate or limited improvement. Interestingly, while activity increased with increasing chain length from ethyl- to pentyl- (**1**–**4**), a significant decrease in activity was observed for hexyl- (**5**) and octyl- (**6**) derivatives. These results indicate that excessive hydrophobicity, long chains, or the presence of multiple aromatic rings may negatively affect cellular performance under inflammatory stress conditions. On the other hand, it is understood that ibuprofen-skeleton amide derivatives with moderate lipophilicity and cyclic flexibility or containing heteroatoms exhibit better preserved cell viability under inflammatory stress due to these substituent groups, rather than solely due to their steric size.

#### Evaluation of Pro-inflammatory Cytokine Production in LPS-Stimulated Macrophages

Based on MTT screening in the presence of LPS-induced inflammatory stress (Figure 2), the compounds that exhibited the highest level of cell viability were selected for further mechanistic evaluation. Compounds **4**, **10**, **11**, and **15** were therefore chosen for further analysis of inflammatory mediator production.

Stimulation of RAW 264.7 macrophages with LPS caused a pronounced increase in IL-1β, TNF-α, and IL-6 levels compared with the Control group (*p* < 0.001 for all), confirming successful induction of an inflammatory response (Figure 3) (for full numerical data and graphs, please see the SI file pages S27 and S28). Pretreatment with ibuprofen significantly reduced LPS-induced IL-1β, TNF-α, and IL-6 levels compared with the LPS group (*p* < 0.05 for all); however, cytokine levels remained significantly higher than those observed in the control group (*p* < 0.05). For IL-1β, treatment with ibuprofen amide derivatives (compounds **4**, **11**, and **15**) resulted in a significant reduction compared with both the LPS and LPS+ibuprofen groups (*p* < 0.05), whereas compound **10**, another ibuprofen amide derivative, showed a significant decrease relative to the control, LPS, and LPS+ibuprofen groups (*p* < 0.05). Among the tested ibuprofen amide derivatives, compound **15** produced the lowest IL-1β levels and was also significantly different from compound **11** (*p* < 0.05). In terms of TNF-α, all tested ibuprofen amide derivatives significantly decreased cytokine levels compared with the LPS group (*p* < 0.05). Ibuprofen amide derivatives **4**, **11**, and **15** also showed significantly lower TNF-α levels than the LPS+ibuprofen group (*p* < 0.05). In contrast, ibuprofen amide derivative **10** reduced TNF-α levels compared with the control and LPS groups, but its effect did not differ significantly from that observed with ibuprofen. Analysis of IL-6 revealed that ibuprofen amide derivative **4** significantly reduced cytokine levels compared with both the LPS and LPS+ibuprofen groups (*p* < 0.05), reaching values comparable to the control group. Ibuprofen amide derivative **15** similarly decreased IL-6 levels relative to LPS and ibuprofen (*p* < 0.05). Ibuprofen amide derivative **10** significantly reduced IL-6 levels compared with the control and LPS groups and also differed from derivative **4** (*p* < 0.05), while ibuprofen amide derivative **11** showed significant reductions relative to the LPS and LPS+ibuprofen groups and differed from derivative **10** (*p* < 0.05). Overall, the statistical analysis demonstrated significant differences among groups for all three cytokines (*p* < 0.001). The magnitude and pattern of cytokine suppression varied among the tested ibuprofen amide derivatives, indicating derivative-specific anti-inflammatory profiles. Overall, these results provide direct biochemical evidence that selected ibuprofen amide derivatives exert genuine anti-inflammatory activity by suppressing key pro-inflammatory cytokines in LPS-stimulated macrophages.

**Figure 3:**
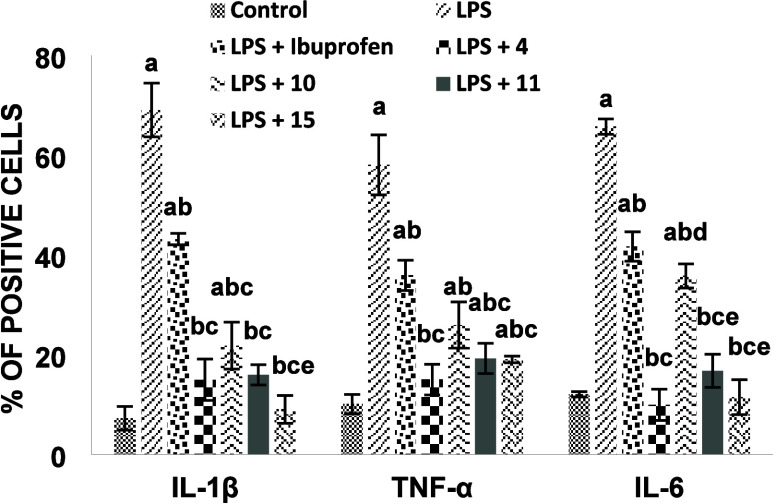
Effects of ibuprofen and selected ibuprofen amide derivatives on LPS-induced pro-inflammatory cytokine production in RAW 264.7 macrophages. RAW 264.7 macrophages were stimulated with lipopolysaccharide (LPS) in the absence or presence of ibuprofen or ibuprofen amide derivatives (compounds **4**, **10**, **11**, and **15**). Intracellular levels of IL-1β, TNF-α, and IL-6 were quantified by flow cytometry. Data are presented as mean ± standard deviation (SD) from four independent experiments. Statistical analysis was performed using one-way ANOVA followed by Tukey’s post hoc test. Statistically significant differences at *P* < 0.05: ^a^compared with control; ^b^compared with LPS; ^c^compared with LPS+ibuprofen; ^d^compared with LPS+4; ^e^compared with LPS+10.

#### Antimicrobial and Antifungal Activities

Next, we decided to test the compounds for their antimicrobial activities. New antimicrobial agents are of interest due to increasing antimicrobial resistance across the world.^29^ To this end, the ibuprofen derivatives were evaluated against the pathogens *Escherichia coli* (ATCC 25292), *MRSA* (BAA 43300), and *Candida albicans* (ATCC 10231) using the well diffusion method (for detailed results, see the SI).

Out of all compounds tested, analogues **15** and **16** exhibited the most significant activities (Table 1). Specifically, these compounds showed broad-spectrum activity against all tested pathogens. Compound **15** produced zones of inhibition (ZOI) with diameters of 9.8, 8.6, and 10.6 mm against *E. coli*, *MRSA*, and *C. albicans*, corresponding to MIC_99_ values of 25.17, 37.9, and 18.4 μM, respectively. Compound **16** showed similar potencies with ZOI of 11.6, 19.8, and 12.7 mm (MIC_99_ 24.3, 16.3, and 23.8 μM).

**Table 1:** Zones of Inhibition (ZOI) and MIC_99_ Obtained from Disk Diffusion Measurements of Antimicrobial and Antifungal Activities^a^

	*E. coli*		MRSA		*C. albicans*	
compound	ZOI (mm)	MIC_99_ (μM)	ZOI (mm)	MIC_99_ (μM)	ZOI (mm)	MIC_99_ (μM)
**3**	ND	>100	ND	>100	10.8	44.1 ± 2.1
**8**	ND	>100	9.8	31.8 ± 2.4	ND	>100
**15**	9.8	25.2 ± 1.6	8.6	37.9 ± 2.1	10.6	18.4 ± 1.7
**16**	11.6	24.3 ± 1.3	20	16.3 ± 1.4	12.7	23.8 ± 1.9

a MIC_99_ values are reported as the mean ± SD for compound–strain combinations showing antimicrobial activity. For combinations where no inhibition was observed, the MIC_99_ value is reported as greater than the highest concentration tested (100 μM), indicating an absence of detectable activity within the tested concentration range. ZOI: zone of inhibition; ND: not detected.

Several other compounds showed activity against a single pathogen: (i) Compounds **8**, **12**, and **13** were observed to form small ZOIs (<6 mm) against *E. coli*. (ii) Compound **8** showed inhibition against *MRSA* (9.8 mm ZOI; MIC_99_ 31.8 μM). In addition, compounds **2**, **5**, and **8** were also observed to form small inhibition zones against *MRSA* (<6 mm). Although their activity was minimal at the tested concentrations, these findings suggest the possibility of strain-specific antimicrobial effects at higher concentrations. (iii) Compound **3** exhibited exclusively antifungal activity with a ZOI of 10.8 mm (MIC_99_ 44.1 μM). (iv) Ibuprofen showed no activity in any tested assay.

When considering the structural features of the broad-spectrum compounds **15** and **16**, it is striking that both compounds feature relatively large, cyclic substituents on the amide functionality (NCy_2_ for **15**; N­(CH_2_(2-pyridyl)_2_ for **16**). Interestingly, structurally related compound **11** with only one cyclohexyl substituent (NHCy) or compounds **12** (NBn_2_) and **14** (NBn­(CHMePh)) do not show any antimicrobial activity under the tested conditions. In combination with the significant difference in polarity between **15** and **16** (influencing important parameters such as cell permeability), these data highlight the nontrivial nature of identifying broad-spectrum antimicrobial activity in the investigated chemical space.

#### Biofilm Inhibition

Encouraged by the above-discussed results from the well diffusion method, we tested the synthesized ibuprofen derivatives for their ability to inhibit biofilm formation of *E. coli* ATCC 25292. Staining the obtained biofilms with crystal violet revealed that three of the tested compounds (**2**, **15**, and **16**) showed activity for biofilm inhibition (see Figure 4). In the *E. coli* strain, the inhibition of biofilms was found to occur at rates of 64, 69, and 79% at the highest concentrations (345, 270, and 258 μM) of compounds **2**, **15**, and **16**, respectively.

**Figure 4:**
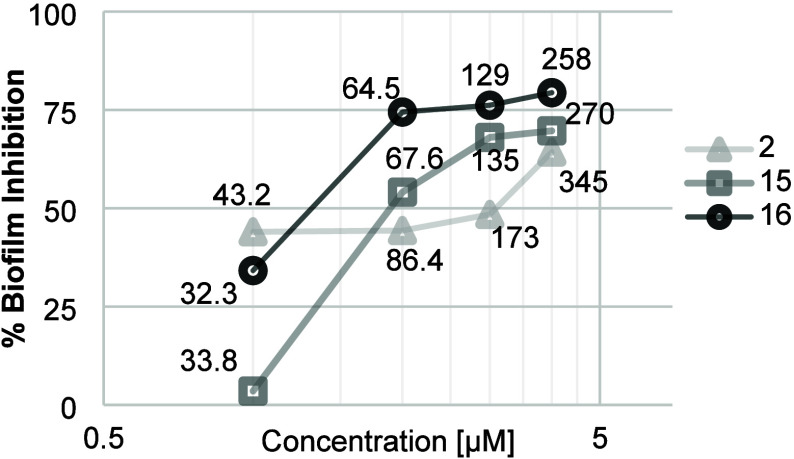
Biofilm inhibition activity in *E. coli* strains. Concentrations expressed in μM correspond to 12.5, 25, 50, and 100 μg/mL for all compounds tested.

## Summary and Conclusions

Overall, this manuscript describes the synthesis of 16 analogues of ibuprofen through a visible-light-promoted, iridium-catalyzed amidation strategy involving the scission of C–N bonds in tertiary amines. Evaluation in diverse biological activities including microbial and biofilm assays suggests interesting biological activities for many of the compounds, with moderate cytotoxic effects up to 100 μM. Anti-inflammatory assays revealed that several derivatives (**2**, **4**, **7**, and **11**–**16**) preserved cell viability better than ibuprofen under LPS-stimulated conditions. Importantly, subsequent analysis of pro-inflammatory cytokine production provided direct biochemical evidence of the anti-inflammatory activity of selected derivatives. This was reflected by the significant suppression of IL-1β, TNF-α, and IL-6 levels in LPS-stimulated RAW 264.7 macrophages. In several cases, the effects observed were more pronounced than those seen with ibuprofen. Screening for antimicrobial activity against *E. coli*, *MRSA*, and *C. albicans* demonstrated notable inhibition by compounds **15** and **16**, which also exhibited significant biofilm inhibition effects. Even though further studies need to be performed to confirm these initial screening results, these data suggest that modifying ibuprofen’s structure is a promising way to develop multifunctional agents.

## Experimental Section

### General Information

All reagents were obtained from commercial suppliers (Alfa Aesar, TCI America, Sigma-Aldrich) and used without further purification unless otherwise specified. Deuterated solvents for NMR analysis were purchased from Cambridge Isotopes or Sigma-Aldrich. The light source employed was a “Blue 5050 72W” LED strip (465 nm, Solid Apollo). This strip was affixed to a glass container, into which the vials for the reactions were placed. 4 mL reaction vials (Chemglass, CG-4904-06) were used for all reactions. NMR measurements were performed on Bruker BioSpin Avance III 400 MHz Digital NMR spectrometers. All spectra were acquired at ambient temperature unless stated otherwise. The residual solvent signal (δ 7.26 ppm for CDCl_3_) was used as the internal reference for ^1^H NMR, while chemical shifts in ^13^C­{H} spectra were referenced to the CDCl_3_ resonance at δ 77.0 ppm.

### Synthesis

Synthesis and isolation of the tested compounds were carried out in analogy to the previously reported procedure.^18^

A solution of the tertiary amine (0.27 mmol, 1.0 equiv) in MeCN (3 mL) was prepared, to which ibuprofen (0.41 mmol, 1.5 equiv), CF_3_SO_2_Na (0.41 mmol, 0.063 g, 1.5 equiv), and [Ir­(dF­(CF_3_)­ppy)_2_(dtbbpy)]­PF_6_ (0.0027 mmol, 0.003 g, 1.0 mol %) were added under an air atmosphere. The vial was sealed with a Teflon-lined cap, and the mixture was stirred at room temperature while being irradiated with blue LEDs for 48 h. Reaction progress was checked by TLC. Upon completion, the solvent was evaporated under reduced pressure.

#### (*S*)-*N*,*N*-Diethyl-2-(4-isobutylphenyl)­propanamide (**1**)

Purification solvent: 1:3 EtOAc/hexane, yield 39.5 mg (56%). ^1^H NMR (400 MHz, CDCl_3_) δ 7.22 (d, *J* = 8.1 Hz, 2H), 7.10 (d, *J* = 8.0 Hz, 2H), 3.71 (q, *J* = 7.2 Hz, 1H), 3.53–3.41 (m, 1H), 3.35–3.21 (m, 1H), 3.13 (dd, *J* = 14.8, 7.2 Hz, 1H), 2.44 (d, *J* = 7.2 Hz, 2H), 1.91–1.79 (m, 1H), 1.50 (d, *J* = 7.2 Hz, 3H), 0.93–0.83 (m, 12H) ppm. The spectral data were in agreement with literature data.^18^

#### (*S*)-2-(4-Isobutylphenyl)-*N*,*N*-dipropylpropanamide (**2**)

Purification solvent: 1:4 EtOAc/hexane, yield 52.3 mg (67%). ^1^H NMR (400 MHz, CDCl_3_) δ 7.16 (d, *J* = 8.1 Hz, 2H), 7.07 (d, *J* = 8.1 Hz, 2H), 3.81 (q, *J* = 6.8 Hz, 1H), 3.41 (ddd, *J* = 13.4, 9.3, 6.2 Hz, 1H), 3.26–3.08 (m, 2H), 3.04–2.95 (m, 1H), 2.43 (d, *J* = 7.2 Hz, 2H), 1.83 (sept, *J* = 6.8 Hz, 1H), 1.56–1.45 (m, 4H), 1.42 (d, *J* = 6.9 Hz, 3H), 0.94–0.76 (m, 12H) ppm. The spectral data were in agreement with literature data.^18^

#### (*S*)-*N*,*N*-Dibutyl-2-(4-isobutylphenyl)­propanamide (**3**)

Purification solvent: 1:1 EtOAc/hexane, yield 57.3 mg (67%). ^1^H NMR (400 MHz, CDCl_3_) δ 7.16 (d, *J* = 8.1 Hz, 2H), 7.07 (d, *J* = 8.1 Hz, 2H), 3.79 (q, *J* = 6.9 Hz, 1H), 3.43 (ddd, *J* = 15.0, 10.4, 5.5 Hz, 1H), 3.27–3.12 (m, 2H), 3.08–2.97 (m, 1H), 2.43 (d, *J* = 7.2 Hz, 2H), 1.83 (sept, *J* = 6.7 Hz, 1H), 1.49–1.38 (m, 7H), 1.31–1.21 (m, 4H), 0.94–0.85 (m, 12H) ppm. The spectral data were in agreement with literature data.^18^

#### (*S*)-2-(4-Isobutylphenyl)-*N*,*N*-dipentylpropanamide (**4**)

Purification solvent: 1:9 EtOAc/hexane, yield 48.5 mg (52%). ^1^H NMR (400 MHz, CDCl_3_) δ 7.16 (d, *J* = 8.1 Hz, 2H), 7.07 (d, *J* = 8.0 Hz, 2H), 3.79 (q, *J* = 6.8 Hz, 1H), 3.48–3.39 (m, 1H), 3.30–3.18 (m, 1H), 3.14 (ddd, *J* = 13.3, 8.7, 6.4 Hz, 1H), 3.00 (ddd, *J* = 15.0, 10.3, 5.0 Hz, 1H), 2.43 (d, *J* = 7.2 Hz, 2H), 1.83 (sept, *J* = 6.6 Hz, 1H), 1.55–1.45 (m, 4H), 1.41 (d, *J* = 6.9 Hz, 3H), 1.35–1.19 (m, 8H), 0.96–0.82 (m, 12H) ppm. The spectral data were in agreement with literature data.^18^

#### (*S*)-*N*,*N*-Dihexyl-2-(4-isobutylphenyl)­propanamide (**5**)

Purification solvent: 1:9 EtOAc/hexane, yield 68.1 mg (70%). ^1^H NMR (400 MHz, CDCl_3_) δ 7.16 (d, *J* = 8.1 Hz, 2H), 7.06 (d, *J* = 8.1 Hz, 2H), 3.79 (q, *J* = 6.8 Hz, 1H), 3.49–3.38 (m, 1H), 3.30–3.19 (m, 1H), 3.14 (ddd, *J* = 13.3, 8.9, 6.2 Hz, 1H), 3.00 (ddd, *J* = 14.9, 10.3, 4.7 Hz, 1H), 2.43 (d, *J* = 7.2 Hz, 2H), 1.84 (sept, *J* = 6.7 Hz, 1H), 1.55–1.45 (m, 4H), 1.41 (d, *J* = 6.8 Hz, 3H), 1.31–1.16 (m, 12H), 0.94–0.83 (m, 12H) ppm. The spectral data were in agreement with literature data.^18^

#### (*S*)-2-(4-Isobutylphenyl)-*N*,*N*-dioctylpropanamide (**6**)

Purification solvent: 1:9 EtOAc/hexane, yield 77.5 mg (69%). ^1^H NMR (400 MHz, CDCl_3_) δ 7.15 (d, *J* = 8.1 Hz, 2H), 7.06 (d, *J* = 8.1 Hz, 2H), 3.79 (q, *J* = 6.9 Hz, 1H), 3.48–3.39 (m, 1H), 3.28–3.18 (m, 1H), 3.18–3.09 (m, 1H), 2.99 (ddd, *J* = 15.0, 10.4, 4.8 Hz, 1H), 2.43 (d, *J* = 7.2 Hz, 2H), 1.84 (sept, *J* = 6.7 Hz, 1H), 1.54–1.44 (m, 4H), 1.41 (d, *J* = 6.8 Hz, 3H), 1.32–1.18 (m, 20H), 0.94–0.83 (m, 12H) ppm. The spectral data were in agreement with literature data.^18^

#### (*S*)-2-(4-Isobutylphenyl)-1-morpholinopropan-1-one (**7**)

Purification solvent: 1:1 EtOAc/hexane, yield 55.1 mg (74%). ^1^H NMR (400 MHz, CDCl3) δ 7.22 (d, *J* = 8.1 Hz, 2H), 7.10 (d, *J* = 8.1 Hz, 2H), 4.05–3.95 (m, 2H), 3.84–3.79 (m, 2H), 3.77–3.69 (m, 5H), 2.45 (d, *J* = 7.2 Hz, 2H), 1.84 (sept, *J* = 6.7 Hz, 1H), 1.51 (d, *J* = 7.2 Hz, 3H), 0.89 (d, *J* = 6.6 Hz, 6H) ppm. The spectral data were in agreement with literature data.^18^

#### (*S*)-2-(4-Isobutylphenyl)-1-(piperidin-1-yl)­propan-1-one (**8**)

Purification solvent: 1:3 EtOAc/hexane, yield 35.4 mg (48%). ^1^H NMR (400 MHz, CDCl_3_) δ 7.22 (d, *J* = 8.1 Hz, 2H), 7.11 (d, *J* = 8.1 Hz, 2H), 4.04–3.99 (m, 1H), 3.75–3.70 (m, 1H), 3.62–3.58 (m, 1H), 2.63 (d, *J* = 7.6 Hz, 1H), 2.45 (d, *J* = 7.2 Hz, 2H), 2.26–2.19 (m, 1H), 1.84 (sept, *J* = 6.7 Hz, 1H), 1.80–1.58 (m, 6H), 1.51 (d, *J* = 7.2 Hz, 3H), 0.90 (d, *J* = 6.6 Hz, 6H) ppm. The spectral data were in agreement with literature data.^18^

#### (*S*)-1-(10,11-Dihydro-5*H*-dibenzo­[*b*,*f*]­azepin-5-yl)-2-(4-isobutylphenyl)­propan-1-one (**9**)

Purification solvent: 1:5 EtOAc/hexane, yield 22.6 mg (30%). ^1^H NMR (400 MHz, CDCl_3_) δ 7.39–7.33 (m, 2H), 7.21–7.09 (m, 4H), 7.07–6.99 (m, 2H), 6.90 (d, *J* = 8.0 Hz, 2H), 6.69 (d, *J* = 8.0 Hz, 2H), 4.01 (q, *J* = 6.8 Hz, 1H), 2.87–2.76 (m, 1H), 2.52–2.43 (m, 2H), 2.40 (d, *J* = 5.9 Hz, 2H), 2.37–2.24 (m, 2H), 1.50 (d, *J* = 6.9 Hz, 3H), 0.88 (d, *J* = 6.6 Hz, 6H) ppm. ^13^C NMR (101 MHz, CDCl3) δ 173.7, 141.2, 140.9, 140.2, 138.5, 137.5, 135.1, 130.3, 130.1, 129.3, 128.9, 128.5, 128.3, 128.0, 127.5, 127.4, 126.9, 126.4, 44.9, 42.8, 30.2, 29.9, 22.2 ppm. IR (KBr): 2952, 2927, 2867, 1667, 1488, 1353, 1266, 745 cm^–1^. GC-MS: 383.3 (M+), 195.2, 161.2, 119.1, 43.1. Elemental analysis: anal. cal. for C_27_H_29_NO; C, 84.55%; H, 7.62%; N, 3.65%. Found: C, 84.52%; H, 7.64%; N, 3.66%.

#### (*S*)-*N*-Ethyl-2-(4-isobutylphenyl)-*N*-phenylpropanamide (**10**)

Purification solvent: 1:4 EtOAc/hexane, yield 32.5 mg (39%). ^1^H NMR (400 MHz, CDCl_3_) δ 7.45–6.75 (m, 9H), 3.83–3.63 (m, 2H), 3.54 (q, *J* = 6.7 Hz, 1H), 2.43 (d, *J* = 6.7 Hz, 2H), 1.89–1.76 (m, 1H), 1.38 (d, *J* = 6.6 Hz, 3H), 1.09 (t, *J* = 6.8 Hz, 3H), 0.91 (d, *J* = 6.3 Hz, 6H) ppm. ^13^C NMR (101 MHz, CDCl3) δ 173.6, 142.0, 139.8, 139.3, 129.3, 129.0, 127.7, 127.2, 45.0, 44.2, 43.1, 30.2, 22.4, 12.9 ppm. IR (KBr): 2955, 2868, 1655, 1494, 1394, 1243, 1132, 699 cm^–1^. GC-MS: 309.2 (M+), 281.2, 161.1, 119.1, 91.0, 32.0. Elemental analysis: anal. cal. for C_21_H_27_NO; C, 81.51%; H, 8.79%; N, 4.53%. Found: C, 81.44%; H, 8.78%; N, 4.54%.

#### (*S*)-*N*-Cyclohexyl-2-(4-isobutylphenyl)-*N*-methylpropanamide (**11**)

Purification solvent: 1:3 EtOAc/hexane, yield 33.3 mg (41%). ^1^H NMR (400 MHz, CDCl_3_) δ 7.21–6.95 (m, 4H), 3.94–3.73 (m, 1H), 3.68–3.41 (m, 1H), 2.78 (d, *J* = 7.3 Hz, 1H), 2.68 (d, *J* = 7.3 Hz, 1H), 2.43 (t, *J* = 7.2 Hz, 2H), 1.89–1.59 (m, 4H), 1.61–1.09 (m, 9H), 0.96–0.80 (m, 7H) ppm. ^13^C NMR (101 MHz, CDCl3) δ 173.3, 173.2, 140.0, 139.9, 139.9, 139.3, 129.4, 126.9, 56.1, 52.5, 45.0, 44.9, 43.5, 30.8, 30.2, 30.1, 29.3, 27.5, 25.6, 22.3, 22.2, 20.8 ppm. IR (KBr): 2927, 2855, 1637, 1451, 1258, 1061, 847 cm^–1^. GC-MS: 301.3 (M+), 188.1, 161.1, 140.1, 83.1, 32.0. Elemental analysis: anal. cal. for C_20_H_31_NO; C, 79.68%; H, 10.36%; N, 4.65%. Found: C, 79.65%; H, 10.42%; N, 4.62%.

#### (*S*)-*N*,*N*-Dibenzyl-2-(4-isobutylphenyl)­propanamide (**12**)

Purification solvent: 1:9 EtOAc/hexane, yield 37.4 mg (36%). ^1^H NMR (400 MHz, CDCl_3_) δ 7.41 – 6.95 (m, 14H), 5.07 (d, J = 14.8 Hz, 1H), 4.58 (d, J = 17.1 Hz, 1H), 4.22 (dd, J = 15.9, 10.2 Hz, 2H), 3.90 (dd, J = 13.3, 6.6 Hz, 1H), 2.48 (d, J = 7.1 Hz, 2H), 1.92 – 1.77 (m, 1H), 1.52 (d, J = 6.8 Hz, 3H), 0.94 (d, J = 6.6 Hz, 6H) ppm. The spectral data were in agreement with literature data.^30^

#### (*S*)-*N*,*N*-Diisobutyl-2-(4-isobutylphenyl)­propanamide (**13**)

Purification solvent: 1:3 EtOAc/hexane, yield 32.3 mg (38%). ^1^H NMR (400 MHz, CDCl_3_) δ 7.20 (d, J = 8.0 Hz, 2H), 7.08 (d, J = 8.0 Hz, 2H), 3.88 (dd, J = 13.6, 6.7 Hz, 1H), 3.59 (dd, J = 13.3, 7.2 Hz, 1H), 3.27 (dd, J = 14.6, 7.7 Hz, 1H), 2.91 – 2.73 (m, 2H), 2.45 (d, J = 7.1 Hz, 2H), 2.05 – 1.79 (m, 3H), 1.44 (d, J = 6.8 Hz, 3H), 0.96 – 0.70 (m, 18H) ppm. The spectral data were in agreement with literature data.^31^

#### (*S*)-*N*-Benzyl-2-(4-isobutylphenyl)-*N*-((*R*)-1-phenylethyl)­propanamide (**14**)

Purification solvent: 1:5 EtOAc/hexane, yield 37.7 mg (35%). ^1^H NMR (400 MHz, CDCl_3_) δ 7.31 (d, *J* = 7.2 Hz, 1H), 7.23 (d, *J* = 7.8 Hz, 1H), 7.18–7.15 (m, 2H), 7.12 (d, *J* = 8.0 Hz, 2H), 7.09–7.05 (m, 3H), 6.98 (d, *J* = 6.6 Hz, 1H), 6.90 (d, *J* = 2.7 Hz, 1H), 6.60 (d, *J* = 7.4 Hz, 1H), 6.08 (q, *J* = 7.0 Hz, 1H), 5.39 (dd, *J* = 13.8, 6.8 Hz, 1H), 4.59 (d, *J* = 15.2 Hz, 1H), 4.30 (s, 1H), 4.04 (dd, *J* = 13.3, 6.4 Hz, 1H), 3.93 (d, *J* = 15.2 Hz, 1H), 3.64 (q, *J* = 7.0 Hz, 1H), 2.46 (dd, *J* = 14.8, 7.2 Hz, 2H), 1.90–1.81 (m, 1H), 1.49 (d, *J* = 7.0 Hz, 2H), 1.42 (dd, *J* = 11.6, 7.0 Hz, 4H), 0.90 (d, *J* = 6.5 Hz, 6H) ppm. ^13^C NMR (101 MHz, CDCl_3_) δ 175.2, 140.5, 140.5, 140.1, 139.5, 138.3, 129.8, 128.3, 128.2, 128.1, 128.0, 127.3, 127.3, 125.9, 54.7, 45.0, 43.5, 43.3, 22.4, 22.3, 21.3, 18.5 ppm. IR (KBr): 3029, 2955, 2868, 1640, 1408, 1165, 696 cm^–1^; GC-MS: 399.3 (M+), 308.2, 161.1, 105.1, 77.0; Elemental analysis: anal. cal. for C_28_H_33_NO; C, 84.17%; H, 8.32%; N, 3.51%. Found: C, 84.12%; H, 8.33%; N, 3.45%.

#### (*S*)-*N*,*N*-Dicyclohexyl-2-(4-isobutylphenyl)­propanamide (**15**)

Purification solvent: 1:9 EtOAc/hexane, yield 30.9 mg (31%). ^1^H NMR (400 MHz, CDCl_3_) δ 7.12 (d, *J* = 7.9 Hz, 2H), 7.06 (d, *J* = 7.9 Hz, 2H), 3.75 (q, *J* = 6.6 Hz, 1H), 3.53 (t, *J* = 11.3 Hz, 1H), 2.74 (t, *J* = 12.8 Hz, 1H), 2.66–2.52 (m, 2H), 2.43 (d, *J* = 7.2 Hz, 2H), 1.88–1.72 (m, 4H), 1.71–1.41 (m, 6H), 1.37 (d, *J* = 6.8 Hz, 3H), 1.33–1.14 (m, 5H), 1.10–0.91 (m, 2H), 0.85 (dd, *J* = 6.5, 2.1 Hz, 6H), 0.82–0.70 (m, 1H), 0.53 (d, *J* = 10.5 Hz, 1H). ^13^C NMR (101 MHz, CDCl_3_) δ 172.6, 140.6, 139.7, 129.4, 126.9, 57.3, 56.1, 45.0, 44.9, 31.2, 30.2, 30.0, 29.1, 26.7, 26.5, 26.1, 25.7, 25.5, 25.2, 22.2, 22.1, 20.9 ppm. ;IR (KBr): 2926, 2853, 1633, 1452, 990, 715 cm^–1^. GC-MS: 369.3 (M+), 208.2, 161.1, 126.1, 83.1, 55.1. Elemental analysis: anal. cal. for C_25_H_39_NO; C, 81.24%; H, 10.64%; N, 3.79%. Found: C, 81.22%; H, 10.59%; N, 3.72%.

#### (*S*)-2-(4-Isobutylphenyl)-*N*,*N*-bis­(pyridin-2-ylmethyl)­propanamide (**16**)

Purification solvent: 1:4 EtOAc/hexane, yield 46.0 mg (44%). ^1^H NMR (400 MHz, CDCl_3_) δ 8.55 (d, *J* = 4.7 Hz, 1H), 8.45 (d, *J* = 4.7 Hz, 1H), 7.56 (ddd, *J* = 14.8, 7.4, 1.4 Hz, 2H), 7.18–7.10 (m, 5H), 7.04 (d, *J* = 7.9 Hz, 2H), 6.97 (d, *J* = 8.0 Hz, 1H), 5.06 (d, *J* = 15.3 Hz, 1H), 4.82 (d, *J* = 17.5 Hz, 1H), 4.48 (dd, *J* = 16.4, 12.3 Hz, 2H), 3.97 (q, *J* = 6.8 Hz, 1H), 2.42 (d, *J* = 7.2 Hz, 2H), 1.82 (dt, *J* = 13.6, 6.7 Hz, 1H), 1.47 (d, *J* = 6.8 Hz, 3H), 0.89 (d, *J* = 6.6 Hz, 6H) ppm. ^13^C NMR (101 MHz, CDCl_3_) δ 174.8, 149.6, 140.2, 138.5, 136.6, 129.4, 127.0, 120.4, 52.6, 51.4, 44.8, 42.7, 30.0, 22.3, 22.2 ppm. IR (KBr): 3060, 2954, 2927, 2868, 1647, 1590, 1434, 1176, 994, 750 cm^–1^. GC-MS: 387.2 (M+), 295.2, 226.1, 93.1, 43.1. Elemental analysis: anal. cal. for C_25_H_29_N_3_O; C, 77.48%; H, 7.54%; N, 10.84%. Found: C, 77.41%; H, 7.59%; N, 10.76%.

### Cytotoxicity (MTT Assay)^28,32^

The cytotoxicity of ibuprofen and its derivatives was evaluated on mouse macrophage RAW 264.7 cells using a standard MTT assay. The cell line purchased from the American Type Culture Collection (ATCC) was cultured in high-glucose Dulbecco’s Modified Eagle’s Medium (DMEM). The medium was supplemented with 10% fetal bovine serum (FBS) and 1% penicillin–streptomycin. Cells were maintained in a humidified incubator at 37 °C with a 5% CO_2_ atmosphere. For the assay, cells were seeded into a 96-well plate at a density of 5 × 10^3^ cells/well. After a 24 h incubation period to allow for cell attachment, the cells were treated with various concentrations of ibuprofen or its derivatives (up to 1 mM). This treatment was administered for 48 h at 37 °C and 5% CO_2_. Control wells contained cells treated with DMEM. Following the treatment period, the MTT reagent was added to each well and the plate was incubated for 4 h. During this time, mitochondrial enzymes in viable cells metabolized the MTT into purple formazan crystals. To quantify cell viability, formazan was dissolved by adding 100 μL of DMSO to each well, followed by vigorous shaking for 15 min to ensure complete solubilization. The absorbance of the resulting purple solution was measured at 570 nm using a ELISA Reader (Thermo Scientific, Multiscan Go).^28^

Cellviability(%)=(sampleabsorbance/controlabsorbance)×100

### Anti-inflammatory Activity

To determine anti-inflammatory potential, RAW 264.7 cells were seeded at a density of 5 × 10^4^ cells/well in a 96-well plate and incubated at 37 °C with 5% CO_2_ for 24 h. Cells were pretreated with 100 μM concentration of ibuprofen and its derivatives for 2 h and stimulated with 1 μg/mL LPS for 22 h, after which the MTT method was applied to determine cell viability. The MTT reagent (0.5 mg/mL in PBS) was then added to the cells, and they were incubated at 37 °C with 5% CO_2_ for 2–4 h. After the culture supernatants were removed, 100 μL of DMSO was added to each well to dissolve formazan, and the absorbance was measured at 570 nm using a microplate reader. The cell viability assay was performed six times.

### Statistical Analysis

SPSS 12.0 statistical software was applied to process and analyze the data, and the results are expressed as average ± standard deviation. Single-factor ANOVA was used for comparison of variables, and *p ≤ 0.05. ** *p* ≤ 0.01 were considered statistically significant.

### Intracellular IL-1β, TNF-α, and IL-6 Staining and Flow Cytometry Analysis

Cells extracted from 80 were fixed with BD Fix Buffer I (557870) following the BD protocol. Cells were then permeabilized with BD Perm Buffer III (558050) and stained separately with BD PE Mouse Anti-IL-1β, TNF, and IL-6. Cells were then analyzed by flow cytometry BD FACS ARIA III. Four replicates were made for each experimental data.^33^

### Preparation Stock Solutions for Bioassay

The masses of all synthesized compounds (**1**–**16**) were accurately measured, and serial dilutions were prepared as outlined in Table S6 in the Supporting Information file page S27. Each compound was dissolved in 1 mL of ethanol to obtain stock solutions. These stock solutions were employed in the disc diffusion assays, while the prepared dilutions were utilized for determining minimum inhibitory concentrations (MIC) and for conducting biofilm inhibition analyses.

### Preparation of Microorganism Cultures

The antimicrobial potential was evaluated on two bacterial strains and one fungal strain. The reference microbial strains used in this study were obtained from the American Type Culture Collection (ATCC, Manassas, Virginia, USA) and are maintained in the culture collection of Harran University, Department of Molecular Biology and Genetics Laboratory, where they are routinely stored and preserved as stock cultures. The bacterial strains used were *E. coli* ATCC 25292, representing Gram-negative bacteria, and methicillin-resistant *Staphylococcus aureus* (MRSA) BAA 43300, representing Gram-positive bacteria, while the fungal strain tested was *C. albicans* ATCC 10231. Frozen stocks of each microorganism were revived in 4 mL of nutrient broth (NB) for bacterial strains and Sabouraud dextrose broth (SDB) for the fungal strain and then incubated at 37 °C overnight. Prior to analysis, cultures were adjusted to a 0.5 McFarland turbidity standard.

### Antimicrobial Activity Analysis

Antibacterial and antifungal activities were assessed using the well diffusion method.^34^ First, Mueller–Hinton agar plates were prepared, and wells with a diameter of 6 mm were carefully created using the back of a pipet tip. Bacterial and fungal cultures were adjusted to a turbidity equivalent to the 0.5 McFarland standard and inoculated evenly onto the surface of Mueller–Hinton agar (MHA) plates with cotton swab. Following inoculation, wells were loaded with 50 μL of the prepared ibuprofen derivative stock solutions at the predetermined concentrations. To enhance compound absorption into the agar, plates were first incubated at +4 °C for 15 min, followed by incubation at 37 °C for 24 h. The diameters of the resulting inhibition zones were recorded in millimeters using a digital caliper. Tetracycline and nystatin were employed as positive controls for bacterial and fungal strains, respectively.

### Determination of Minimum Inhibitory Concentrations (MIC_99_)

MIC values were determined using the microbroth dilution method in sterile 96-well microplates. Each well contained 200 μL of Mueller–Hinton Broth (MHB). Twofold serial dilutions of the compounds were prepared along the *y*-axis, followed by the addition of 10 μL of overnight microbial cultures adjusted to 0.5 McFarland turbidity. The negative control wells contained only the medium and microorganism, while the positive controls consisted of tetracycline for bacterial strains and nystatin for the fungal strain. The plates were incubated at 37 °C for 24 h. After incubation, bacterial and fungal growth was evaluated by measuring the absorbance at 600 nm. The MIC_9__9_ value is defined as the lowest extract concentration that inhibits 99% of microbial growth and was calculated according to the formula used in our previous studies. All experiments were performed in triplicate.^35^

$$\mathrm{Inhibition}\;(\%)=[1‐{\mathrm{OD}}_{t\mathrm{est}\;\mathrm{well}}/{\mathrm{OD}}_{\mathrm{corresponding}\;\mathrm{control}\;\mathrm{well}}]\times 100$$

### Antibiofilm Activity^36^

The capacity of the synthesized ibuprofen derivatives to inhibit biofilm formation was evaluated against *E. coli* ATCC 25292. Overnight cultures of each strain were adjusted to the 0.5 McFarland standard and inoculated into the wells of sterile 96-well microplates containing the test compounds at various subinhibitory concentrations. The plates were incubated at 37 °C for 24 h under static conditions.

After incubation, planktonic cells were removed, and wells were gently washed with sterile phosphate-buffered saline (PBS, pH 7.2) to remove nonadherent cells. Plates were air-dried, and the attached biofilms were stained with 0.1% crystal violet. Excess dye was rinsed off with distilled water, and bound dye was solubilized in 95% ethanol. The absorbance was measured at 595 nm using a microplate reader. Wells containing only the microbial inoculum served as the negative control, while tetracycline (for bacteria) and nystatin (for the fungus) were used as positive controls. The percentage of biofilm inhibition was calculated using the following formula:

$$\mathrm{Biofilm}\;\mathrm{Inhibition}\left(\%\right)=\left(\frac{\mathrm{Abs}\left(\mathrm{control}\right)-\mathrm{Abs}\left(\mathrm{sample}\right)}{\mathrm{Abs}\left(\mathrm{control}\right)}\right)\times 100$$

## Supplementary Material



## Data Availability

The data supporting this article are provided in the Supporting Information.

## Notes

^1^H NMR spectra of literature-known compounds; ^1^H NMR, ^13^C NMR, FTIR, and GC-MS spectra of newly synthesized compounds; and antimicrobial assay images (PDF)
