# Supplementary material for: Anti-inflammatory and Antimicrobial Properties of Ibuprofen Analogues Derived by Photoredox-Catalyzed C–N Scission of Tertiary Amines and Amidation
Source: ACS Omega. 2026 Feb 25;11(9):14860–8. doi: 10.1021/acsomega.5c11131 (PMC12980210; doi:10.1021/acsomega.5c11131)
Supplement: Supplementary file 1 [file ao5c11131_si_001.pdf]

Supporting Information

Anti-inflammatory and anti-microbial properties of ibuprofen analogues derived by photoredox-catalyzed C-N scission of tertiary amines and amidation

Ozgur YILMAZ*[a], Merve DOGAN,[a] Derya YETKIN,[b] Pinar KUCE CEVIK[c] and Marion H. Emmert[d]

[a]Department of Chemistry, Faculty of Sciences, Mersin University, 33343 Mersin, Turkey
[b]Advanced Technology Research and Application Center, Mersin University, Mersin, Turkey
[c]Department of Molecular Biology and Genetic, Faculty of Science and Arts, Harran University, 63290 Sanliurfa, Turkey.
[d]Discovery Chemistry, MRL, Merck & Co., Inc., 770 Sumneytown Pike, West Point, PA 19486, USA.

Email: yilmazozgur@mersin.edu.tr

Table of Contents

General Procedure for Amide synthesis after optimization 3

NMR and GCMS spectra of products.. 3

(S)-*N,N*-diethyl-2-(4-isobutylphenyl)propanamide ($C_{17}H_{27}NO$, 1) 5

(S)-2-(4-isobutylphenyl)-*N,N*-dipropylpropanamide ($C_{19}H_{31}NO$, 2).. 7

(S)-*N,N*-dibutyl-2-(4-isobutylphenyl)propanamide ($C_{21}H_{35}NO$, 3).. 8

(S)-2-(4-isobutylphenyl)-*N,N*-dipentylpropanamide ($C_{23}H_{39}NO$, 4).. 10

(S)-*N,N*-dihexyl-2-(4-isobutylphenyl)propanamide ($C_{25}H_{43}NO$, 5) 12

(S)-2-(4-isobutylphenyl)-*N,N*-dioctylpropanamide ($C_{29}H_{51}NO$, 6) 13

(S)-2-(4-isobutylphenyl)-1-morpholinopropan-1-one ($C_{17}H_{25}NO$, 7)... 14

(S)-2-(4-isobutylphenyl)-1-(piperidin-1-yl)propan-1-one ($C_{18}H_{27}NO$, 8).. 15

(S)-1-(10,11-dihydro-5H-dibenzo[b,f]azepin-5-yl)-2-(4-isobutylphenyl)propan-1-one (9) 16

(S)-*N*-ethyl-2-(4-isobutylphenyl)-*N*-phenylpropanamide (10) 18

(S)-*N*-cyclohexyl-2-(4-isobutylphenyl)-*N*-methylpropanamide (11) 20

(S)-*N,N*-dibenzyl-2-(4-isobutylphenyl)propanamide (12).. 22

(S)-*N,N*-diisobutyl-2-(4-isobutylphenyl)propanamide (13) 23

(S)-*N*-benzyl-2-(4-isobutylphenyl)-*N*-((R)-1-phenylethyl)propanamide (14)................................. 24

(S)-*N,N*-dicyclohexyl-2-(4-isobutylphenyl)propanamide (15) 26

(S)-2-(4-isobutylphenyl)-*N,N*-bis(pyridin-2-ylmethyl)propanamide (16)....................................... 28

ANTI-INFLAMMATORY ACTIVITIES... 30

ANTI-MICROBIAL ACTIVITIES.. 33

General Procedure for Amide synthesis after optimization

NR_3 or HNR_2 (0.27 mmol, 64 µL, 1.0 equiv), 3 mL MeCN, $(Ir[dF(CF_3)ppy]_2(dtbpy))PF_6$ (0.0027 mmol, 0.003 g, 0.01 equiv), ibuprofen (0.41 mmol, 1.5 equiv, 0.085 g), and CF_3SO_2Na (0.41 mmol, 0.063 g, 1.5 equiv) were mixed in a 4 mL glass vial equipped with a stir bar. The vial was sealed and the reaction mixture was stirred for 48 h at room temperature while irradiating with blue LEDs.

To determine crude assay yields by GC, decane or dodecane was added to the reaction mixture. The mixture was sampled by diluting an aliquot with MeCN or EtOAc, followed by filtration, and analysis of the filtrate by GC-FID. Yields were determined by use of a previously established calibration curve.

To determine crude assay yields by quantitative ^1H NMR, the reaction mixture was evaporated. Then, $CDCl_3$ and 1,1,2-trichloroethane or *p*-xylene (as internal standards) were added. The resulting suspension was mixed well, filtered, and analyzed by quantitative ^1H NMR.

NMR and GCMS spectra of products

Literature-known compounds. Graphics of ^1H-NMR spectra are provided below for compounds known in the literature. The data for these ^1H NMR spectra are detailed in the Experimental Section of the manuscript. The data were compared with data in the literature and found to be in agreement; relevant literature references for those compounds are also provided directly in the Experimental Section of the manuscript.

Compounds not known in the literature. ^1H, ^{13}C, GC-MS, FTIR and elemental analysis measurements were obtained for newly synthesized compounds that are unknown in the literature. The data are available in the manuscript's experimental section. Graphics for NMR as well as GC-MS and FTIR spectra are provided below.

(S)-*N,N*-diethyl-2-(4-isobutylphenyl)propanamide ($C_{17}H_{27}NO$, 1)

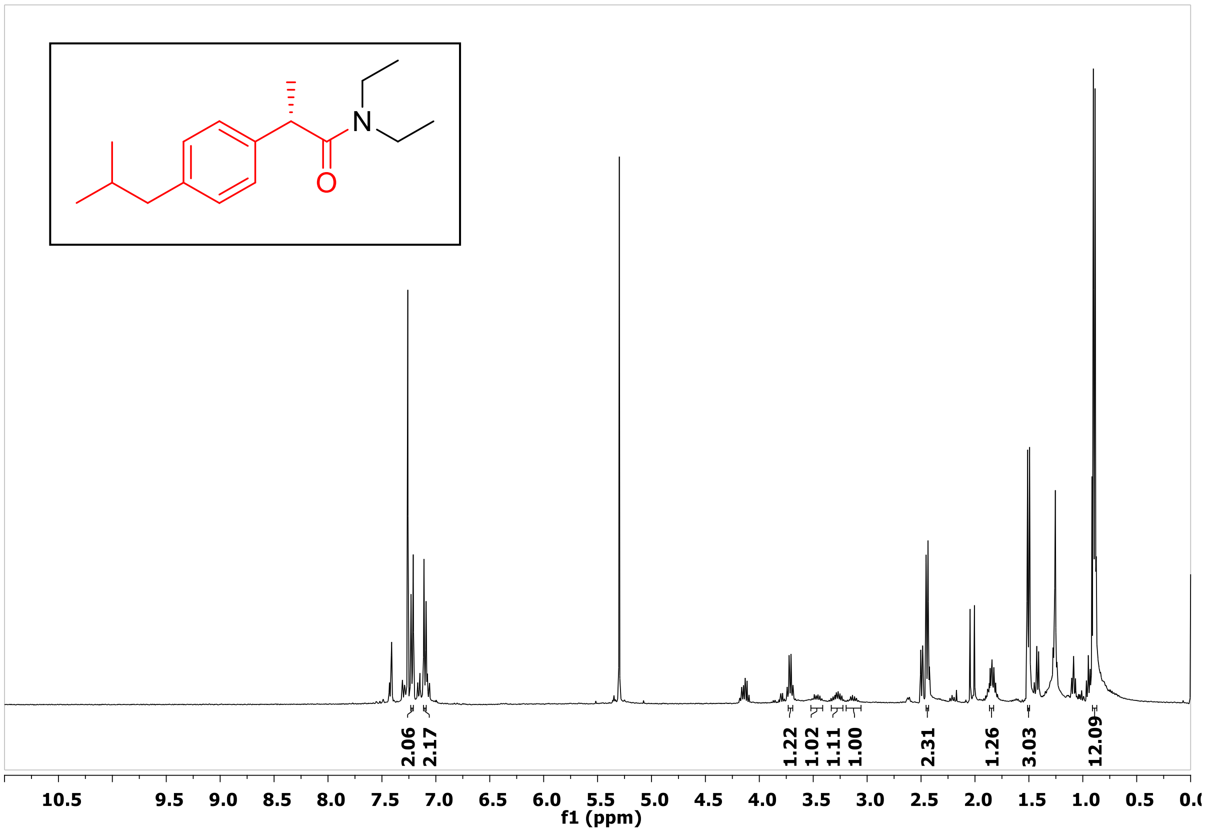

*Figure S1. ^{1}H NMR spectrum of (S)-N,N-diethyl-2-(4-isobutylphenyl)propanamide **(1)** ($CDCl_3$), 400 MHz.*

(S)-2-(4-isobutylphenyl)-*N,N*-dipropylpropanamide ($C_{19}H_{31}NO$, 2)

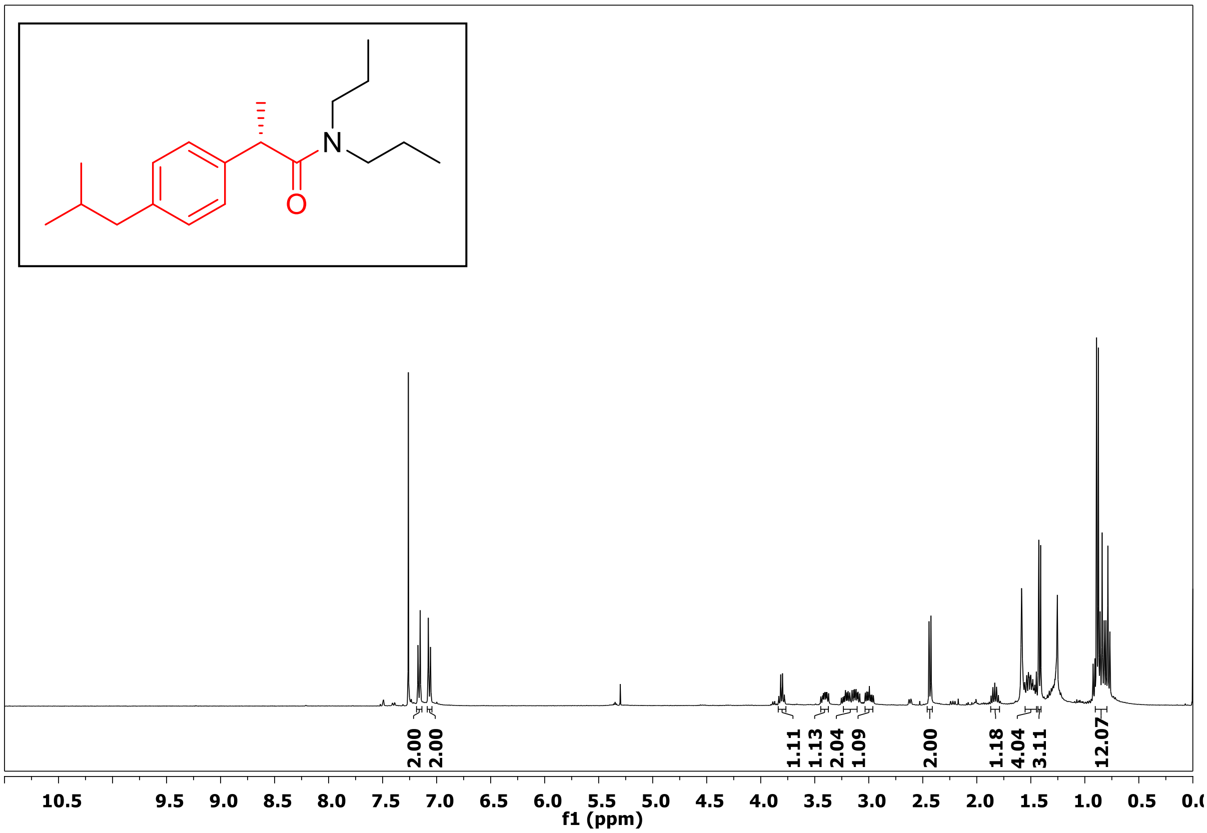

Figure S2. ¹H NMR spectrum of (S)-2-(4-isobutylphenyl)-N,N-dipropylpropanamide ***(2)*** *($CDCl_3$), 400 MHz.*

(S)-*N,N*-dibutyl-2-(4-isobutylphenyl)propanamide ($C_{21}H_{35}NO$, 3)

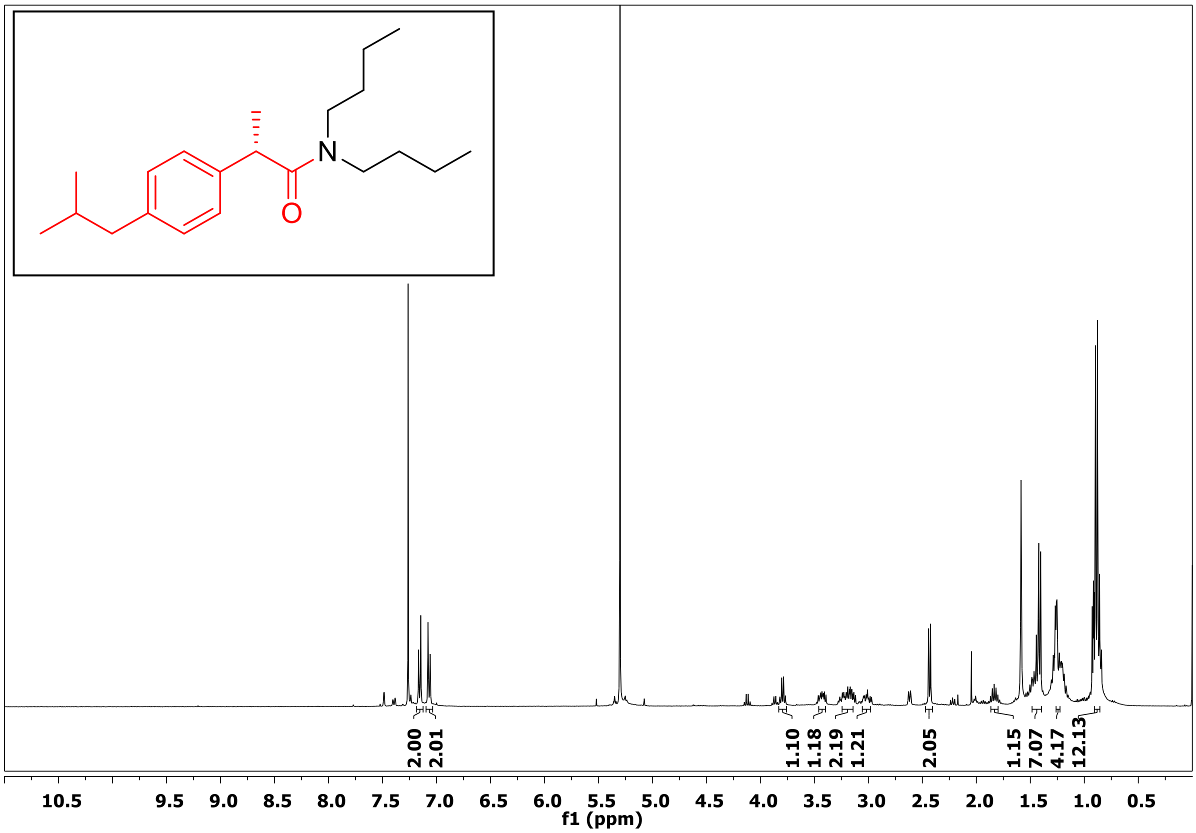

*Figure S3. ^{1}H NMR spectrum of (S)-N,N-dibutyl-2-(4-isobutylphenyl)propanamide **(3)** ($CDCl_3$), 400 MHz.*

(S)-2-(4-isobutylphenyl)-*N,N*-dipentylpropanamide ($C_{23}H_{39}NO$, 4)

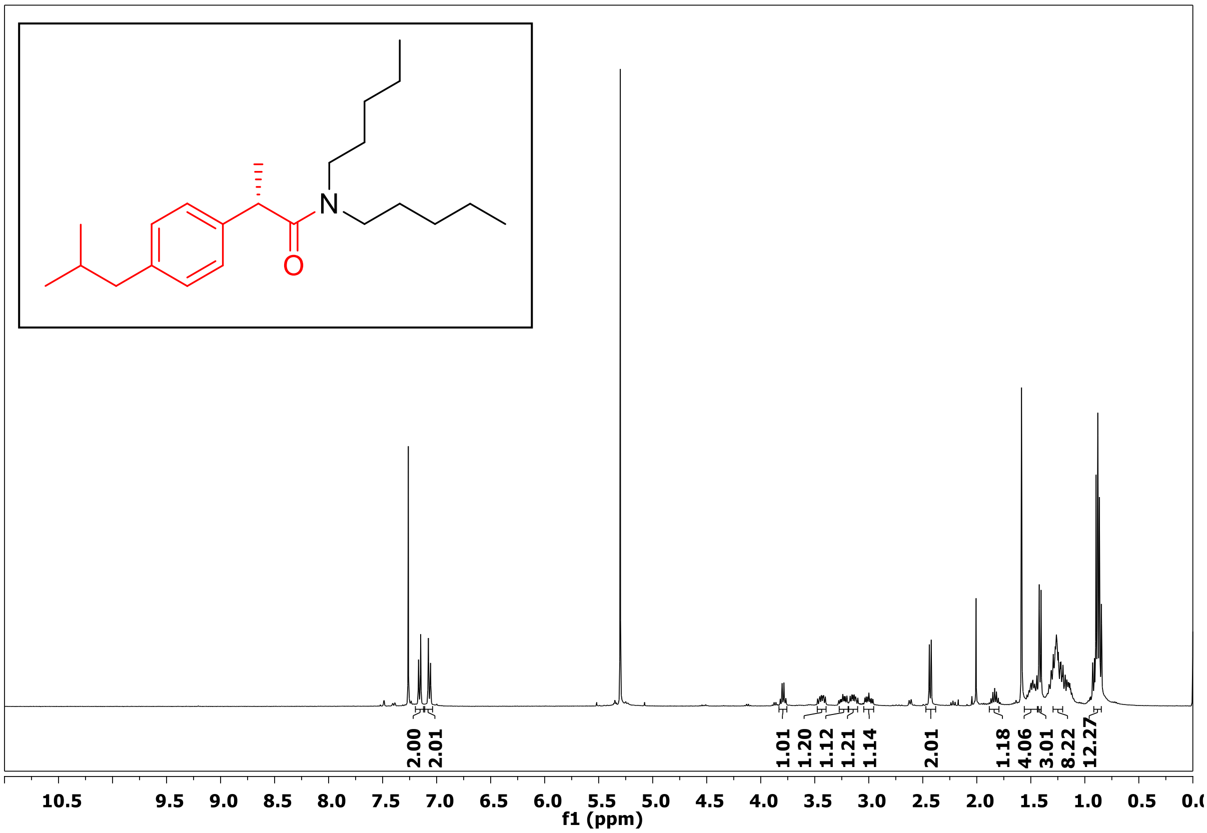

Figure S4. ^{1}H NMR spectrum of (S)-2-(4-isobutylphenyl)-N,N-dipentylpropanamide ***(4)*** *($CDCl_3$), 400 MHz.*

(S)-*N,N*-dihexyl-2-(4-isobutylphenyl)propanamide ($C_{25}H_{43}NO$, 5)

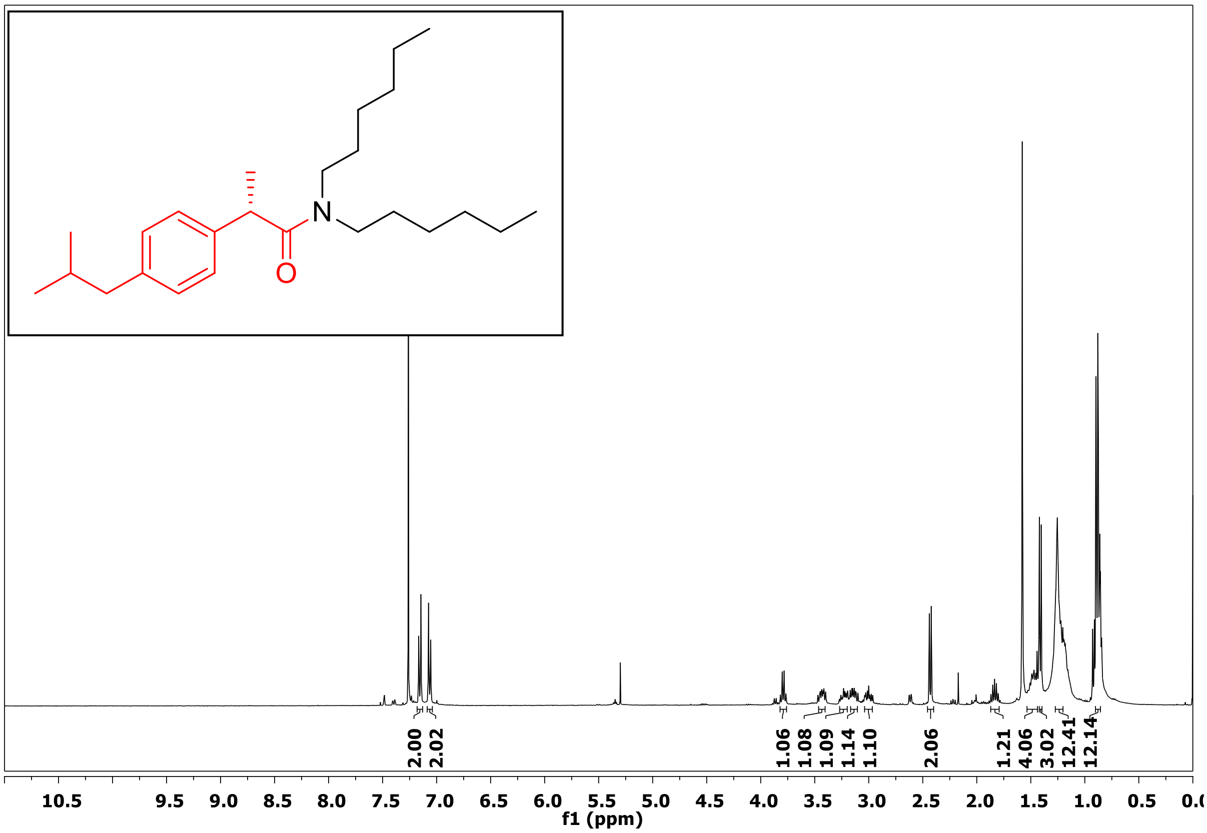

Figure S5. ^{1}H NMR spectrum of (S)-N,N-dihexyl-2-(4-isobutylphenyl)propanamide ***(5)*** *($CDCl_3$), 400 MHz.*

(S)-2-(4-isobutylphenyl)-*N,N*-dioctylpropanamide ($C_{29}H_{51}NO$, 6)

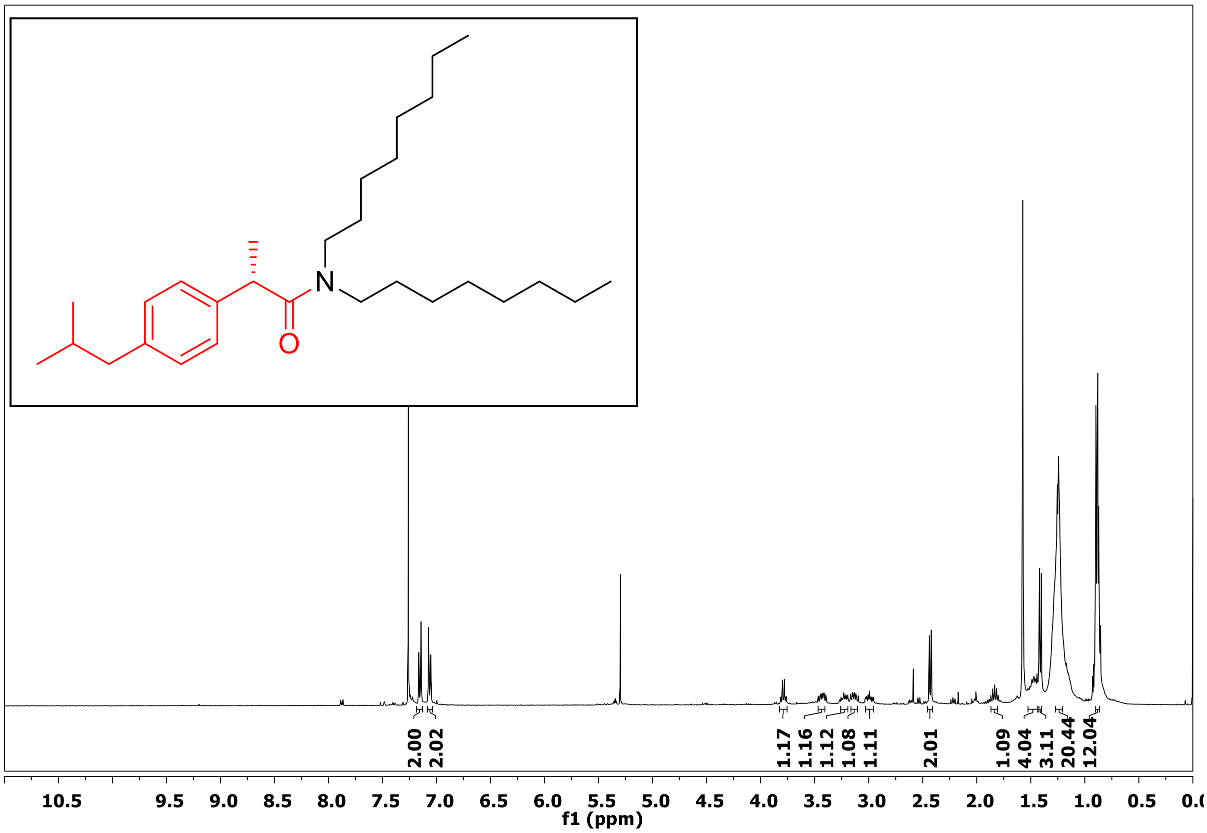

Figure S6. ${}^{1}H$ NMR spectrum of (S)-2-(4-isobutylphenyl)-N,N-dioctylpropanamide ***(6)*** *($CDCl_3$), 400 MHz.*

(S)-2-(4-isobutylphenyl)-1-morpholinopropan-1-one ($C_{17}H_{25}NO$, 7)

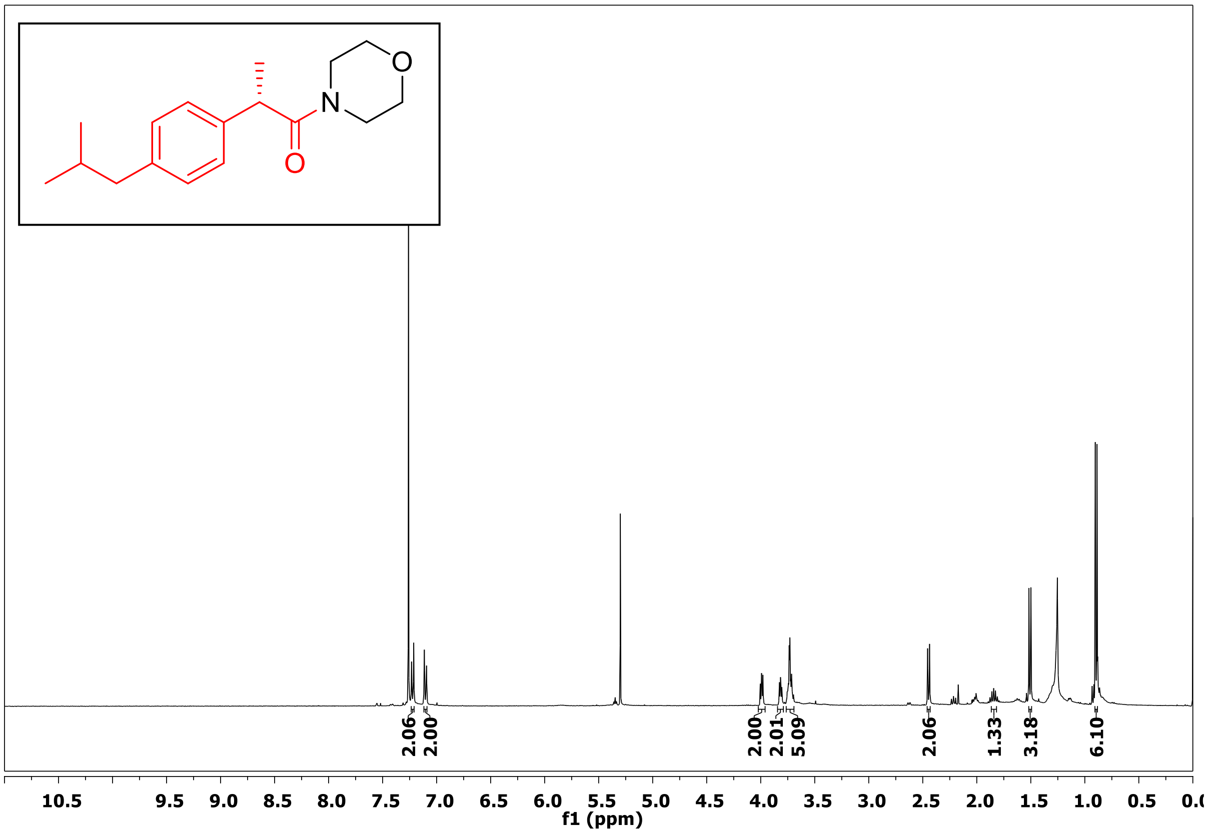

Figure S7. ^{1}H NMR spectrum of (S)-2-(4-isobutylphenyl)-1-morpholinopropan-1-one ***(7)*** *($CDCl_3$), 400 MHz.*

(S)-2-(4-isobutylphenyl)-1-(piperidin-1-yl)propan-1-one ($C_{18}H_{27}NO$, 8)

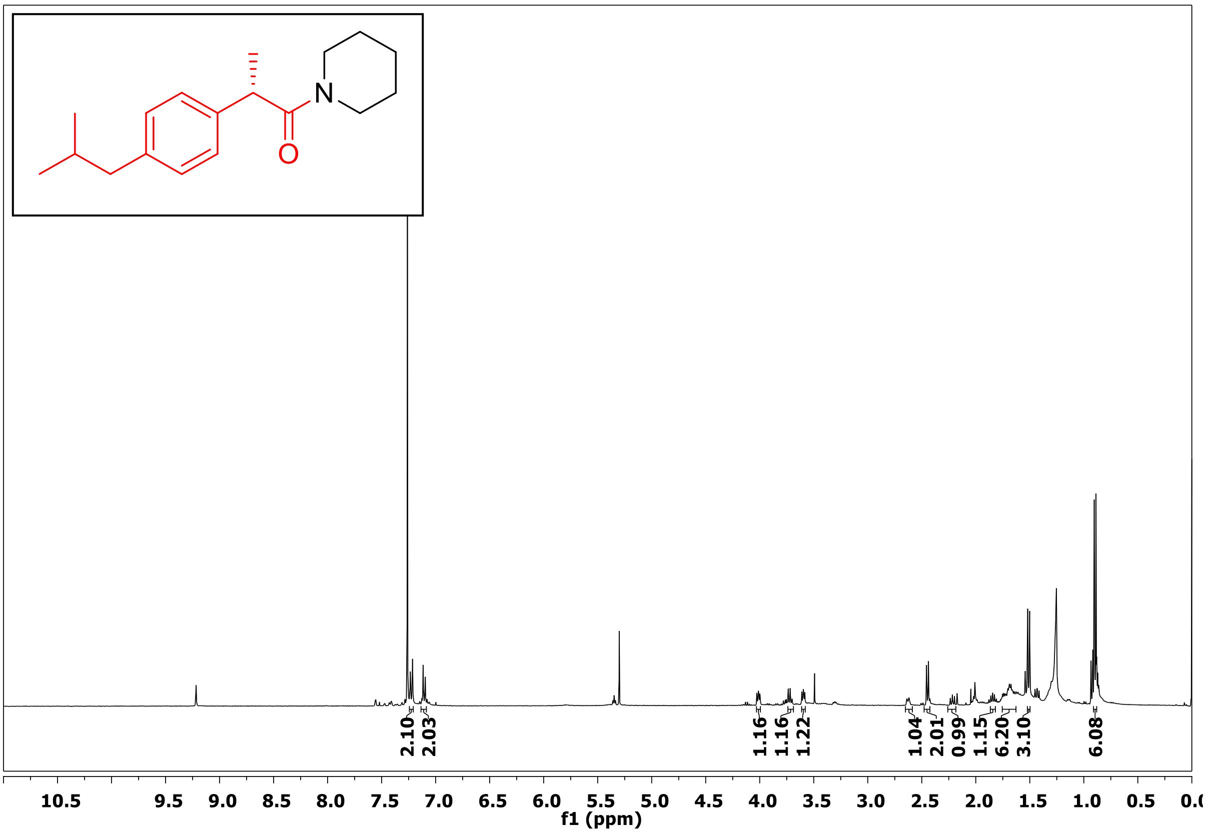

*Figure S8. ^{1}H NMR spectrum of (S)-2-(4-isobutylphenyl)-1-(piperidin-1-yl)propan-1-one **(8)** ($CDCl_3$), 400 MHz.*

(S)-1-(10,11-dihydro-5H-dibenzo[b,f]azepin-5-yl)-2-(4-isobutylphenyl)propan-1-one (9)

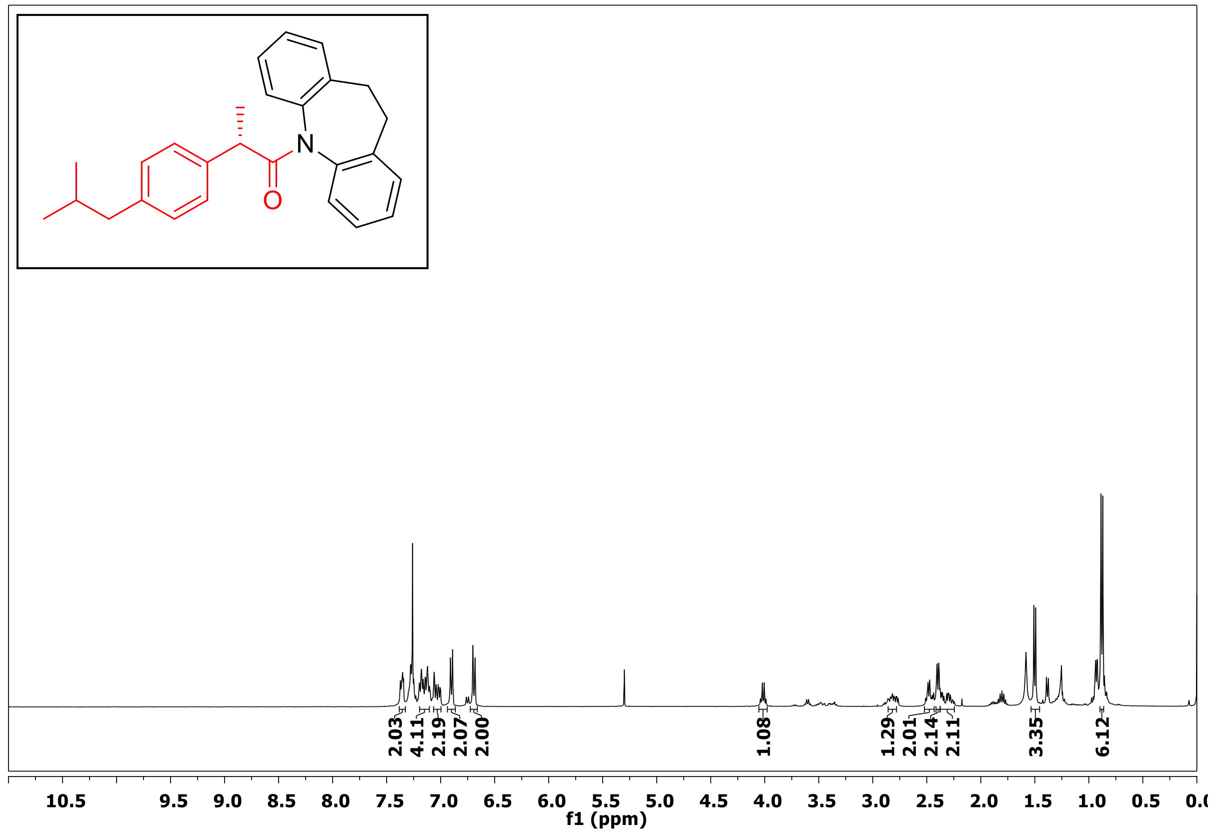

*Figure S9. ^{1}H NMR spectrum of (S)-1-(10,11-dihydro-5H-dibenzo[b,f]azepin-5-yl)-2-(4-isobutylphenyl)propan-1-one **(9)** ($CDCl_3$), 400 MHz.*

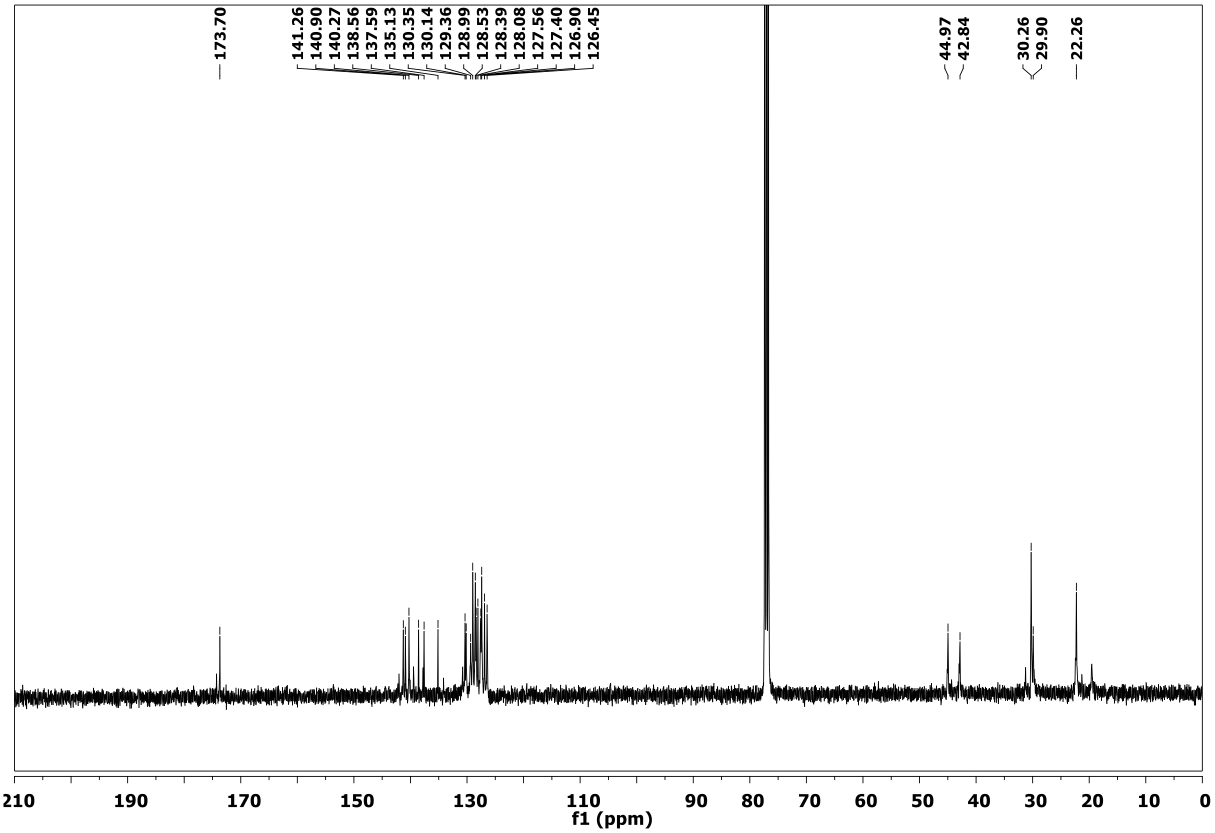

*Figure S10. ^{13}C NMR spectrum of (S)-1-(10,11-dihydro-5H-dibenzo[b,f]azepin-5-yl)-2-(4-isobutylphenyl)propan-1-one **(9)** ($CDCl_3$), 101 MHz.*

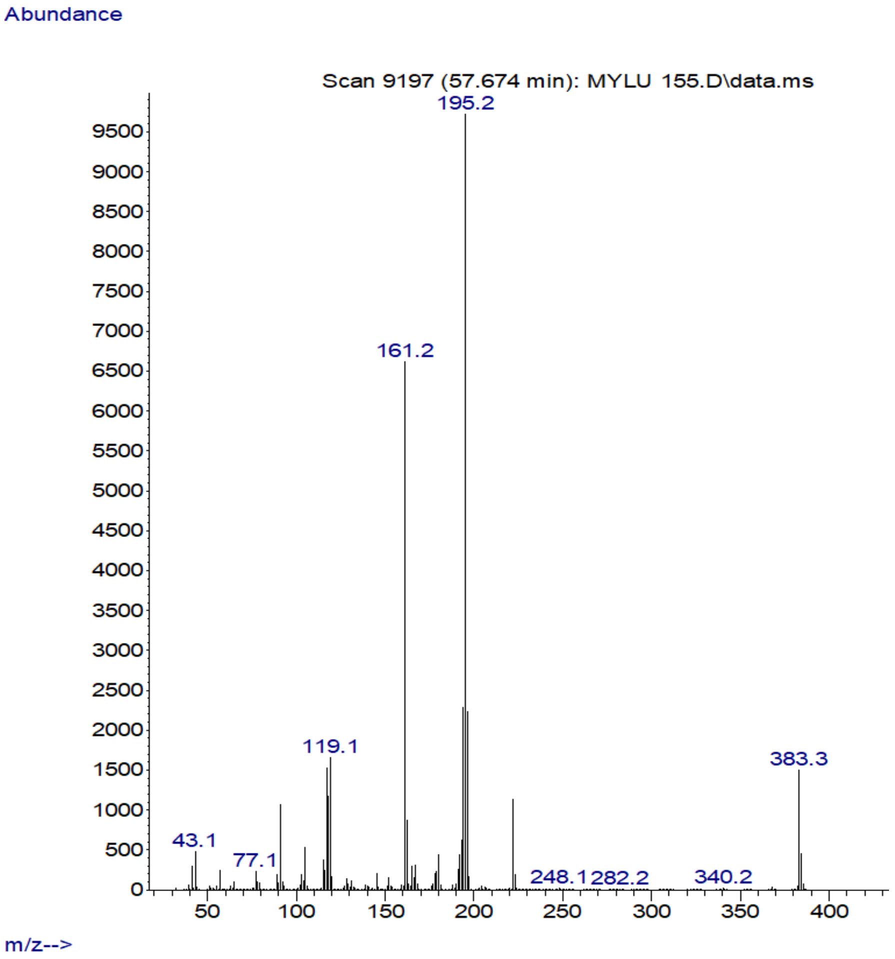

Figure S11. MS trace of (S)-1-(10,11-dihydro-5H-dibenzo[b,f]azepin-5-yl)-2-(4-isobutylphenyl)propan-1-one ***(9)***

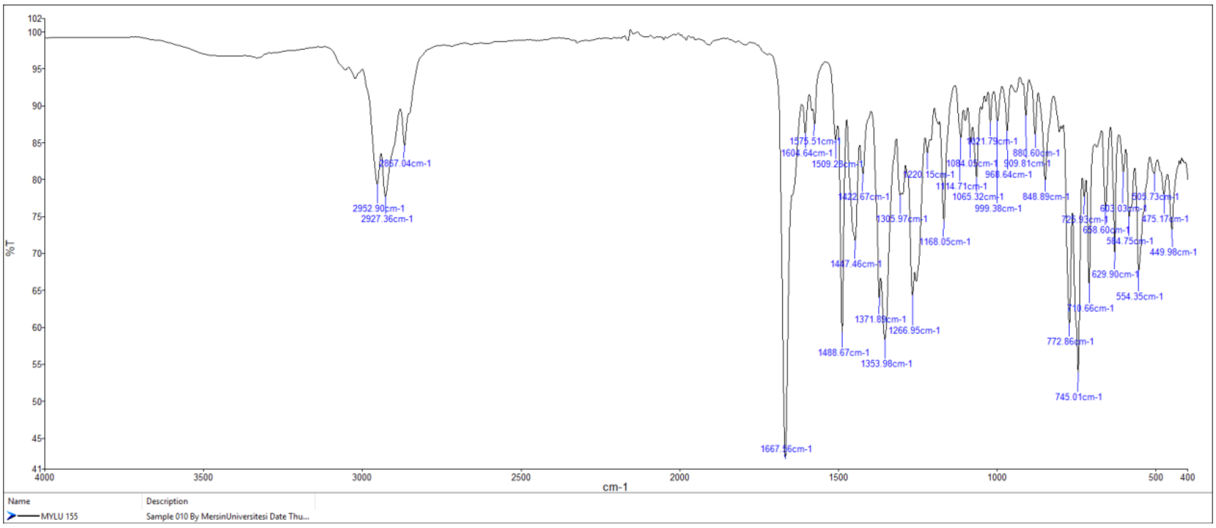

Figure S12. FTIR spectrum of (S)-1-(10,11-dihydro-5H-dibenzo[b,f]azepin-5-yl)-2-(4-isobutylphenyl)propan-1-one ***(9).***

(S)-*N*-ethyl-2-(4-isobutylphenyl)-*N*-phenylpropanamide (10)

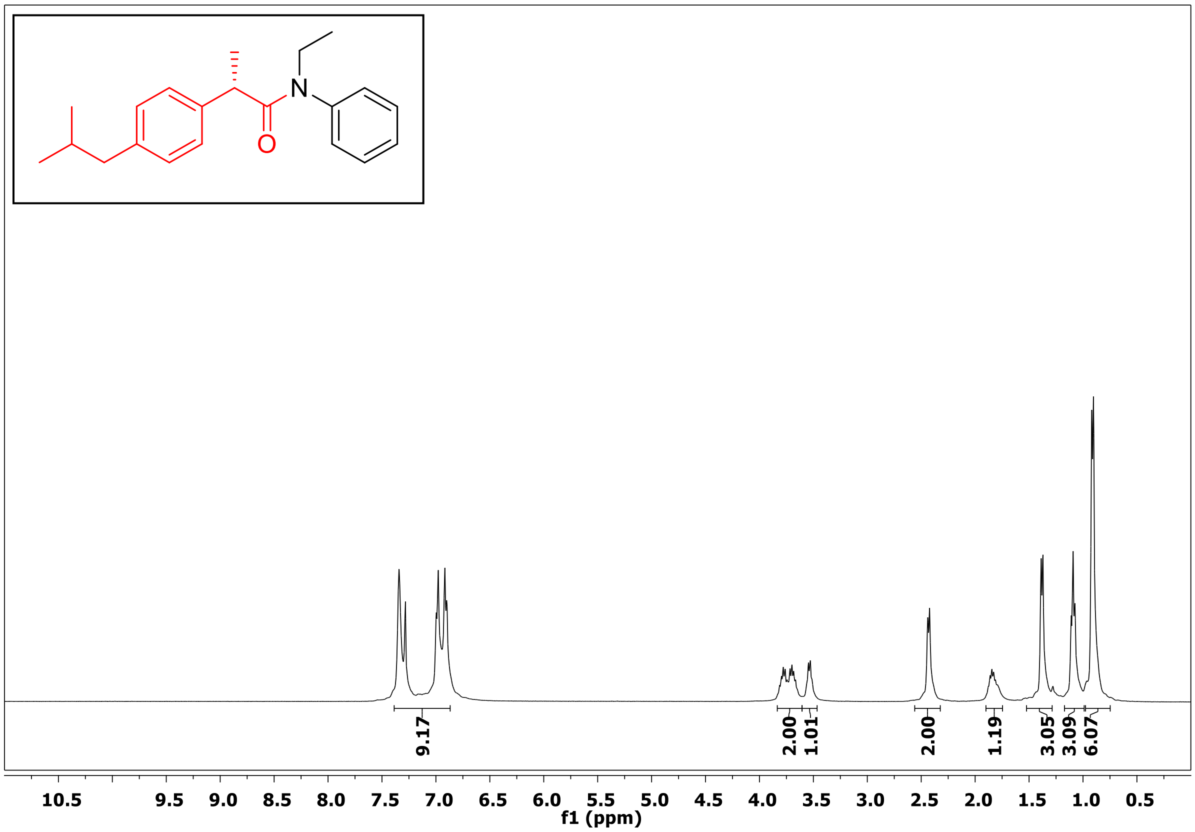

*Figure S13. ¹H NMR spectrum of (S)-N-ethyl-2-(4-isobutylphenyl)-N-phenylpropanamide **(10)** ($CDCl_3$), 400 MHz.*

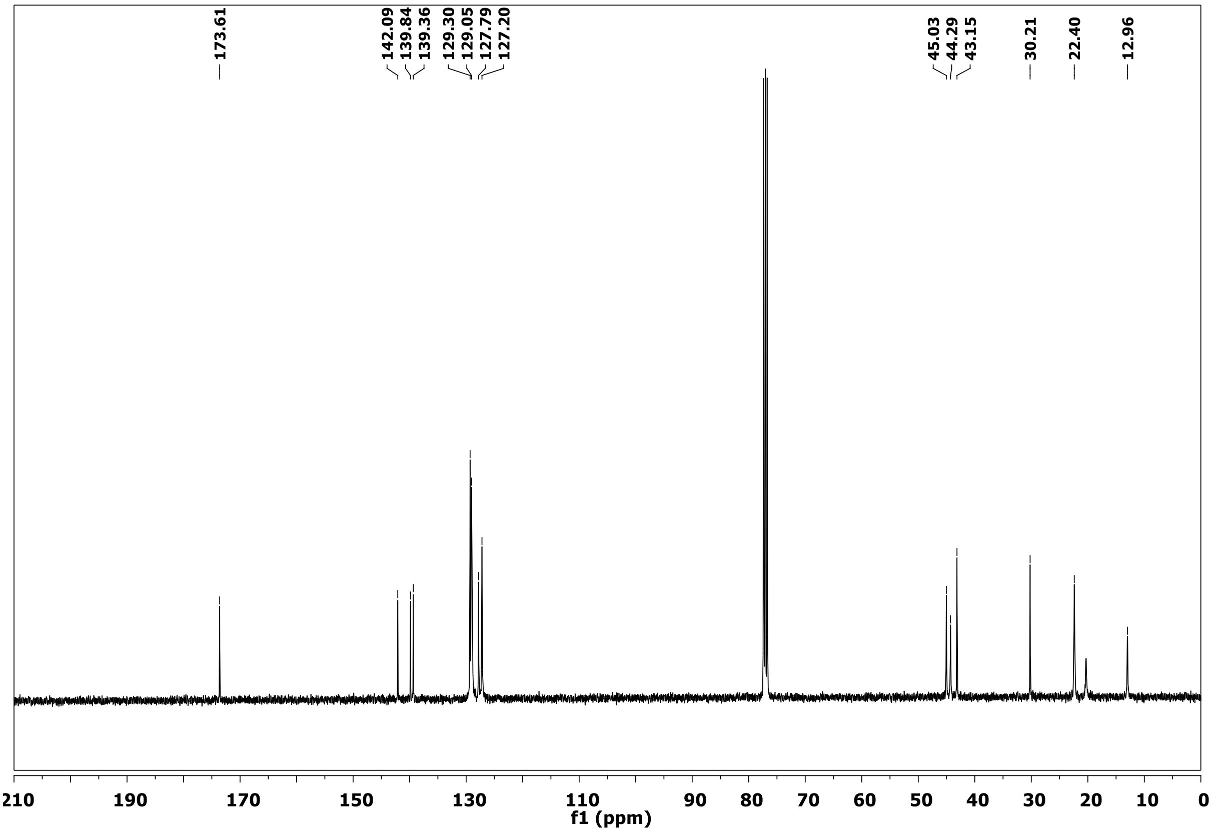

*Figure S14. ¹³C NMR spectrum of (S)-N-ethyl-2-(4-isobutylphenyl)-N-phenylpropanamide **(10)** ($CDCl_3$), 101 MHz.*

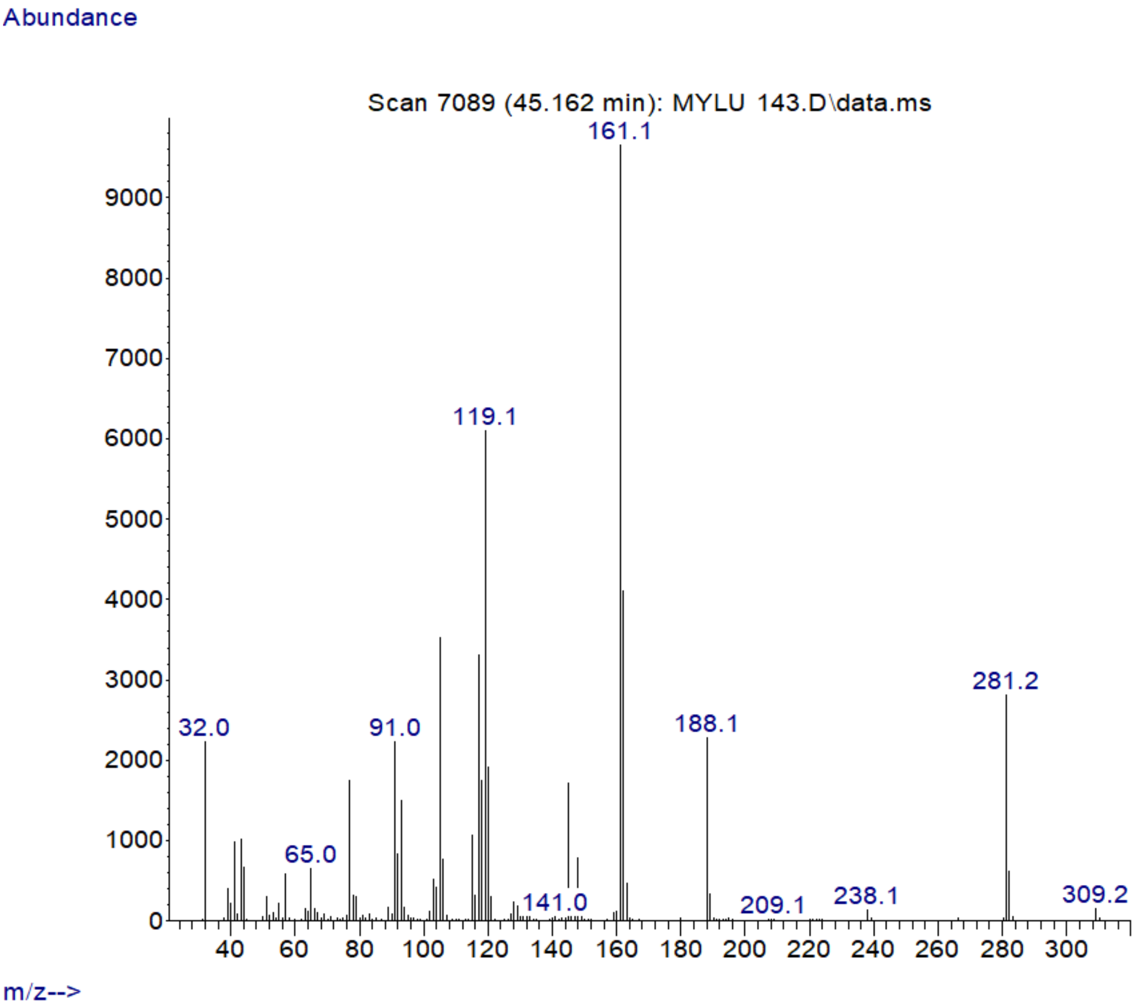

*Figure S15. MS trace of (S)-N-ethyl-2-(4-isobutylphenyl)-N-phenylpropanamide **(10).***

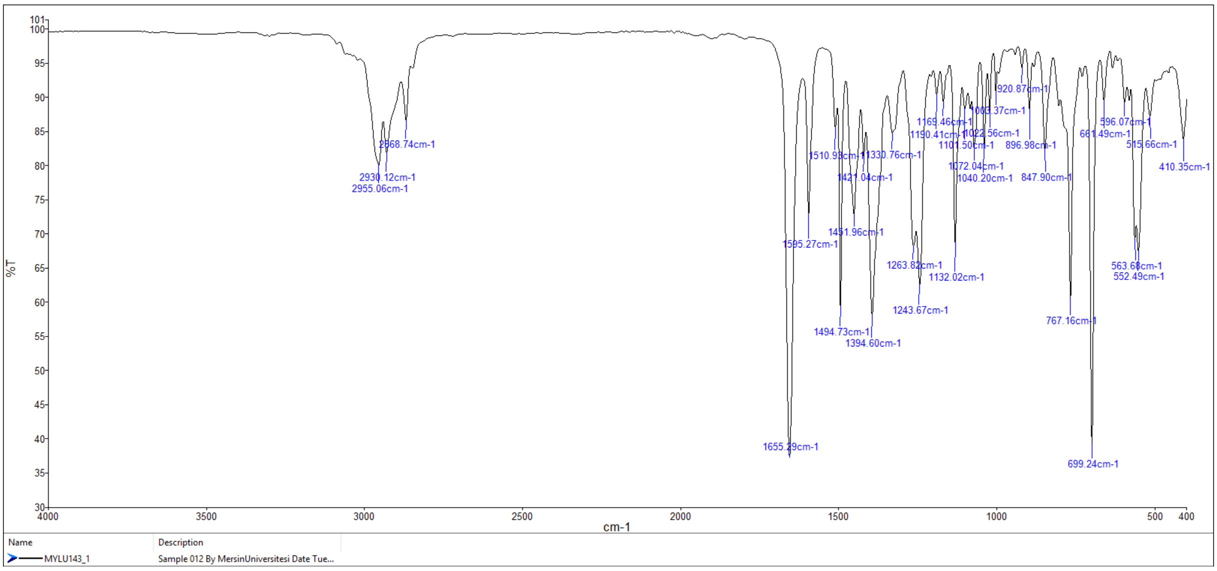

*Figure S16. FTIR spectrum of (S)-N-ethyl-2-(4-isobutylphenyl)-N-phenylpropanamide **(10).***

(S)-*N*-cyclohexyl-2-(4-isobutylphenyl)-*N*-methylpropanamide (11)

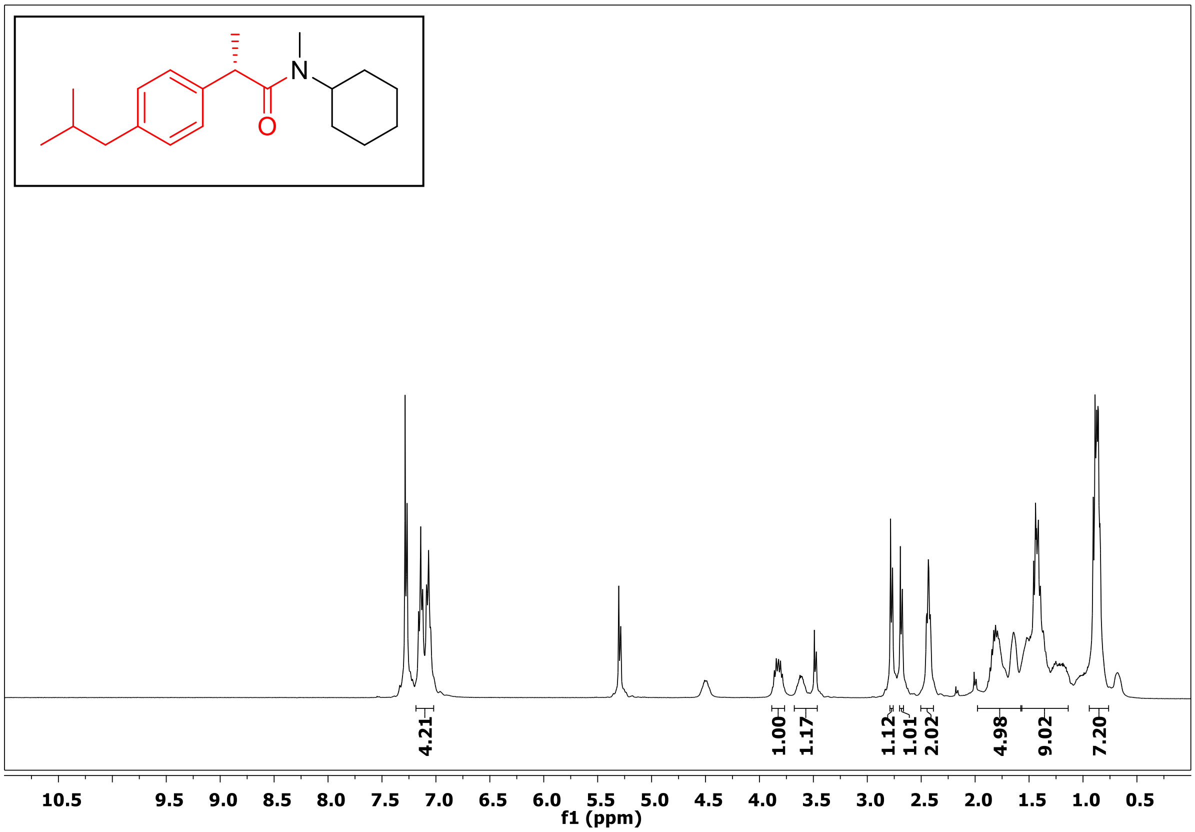

Figure S17. ^{1}H NMR spectrum of (S)-N-cyclohexyl-2-(4-isobutylphenyl)-N-methylpropanamide ***(11)*** *($CDCl_3$), 400 MHz.*

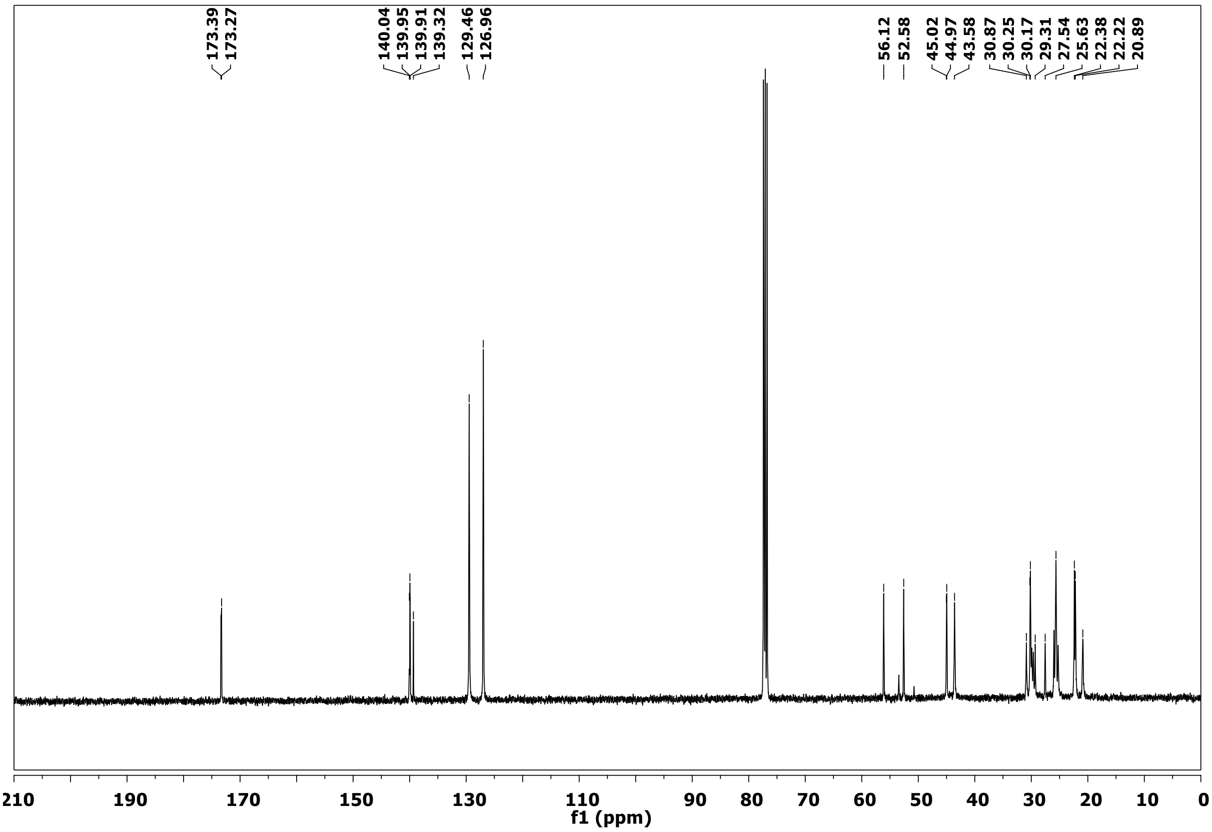

Figure S18. ^{13}C NMR spectrum of (S)-N-cyclohexyl-2-(4-isobutylphenyl)-N-methylpropanamide ***(11)*** *($CDCl_3$), 101 MHz.*

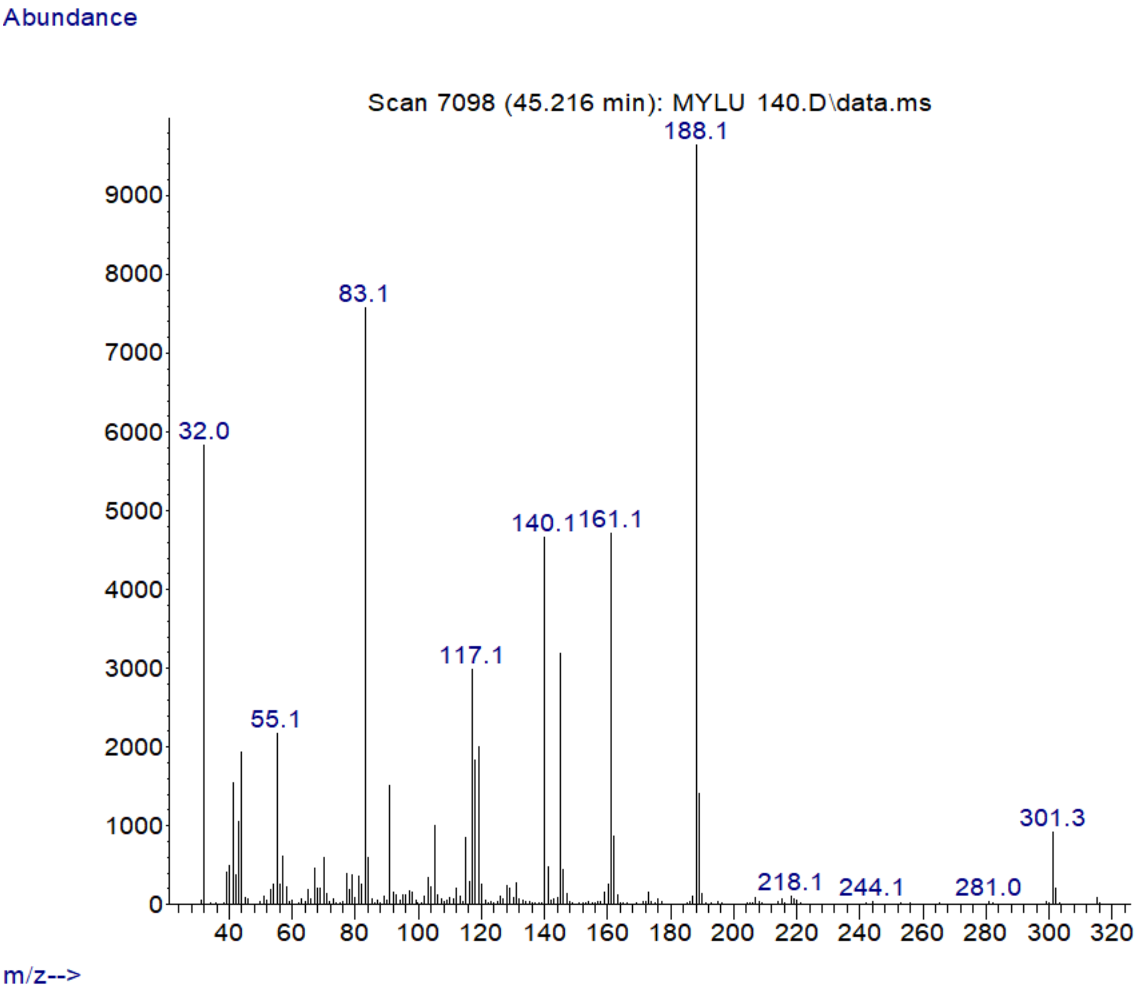

Figure S19. MS trace of (S)-N-cyclohexyl-2-(4-isobutylphenyl)-N-methylpropanamide ***(11).***

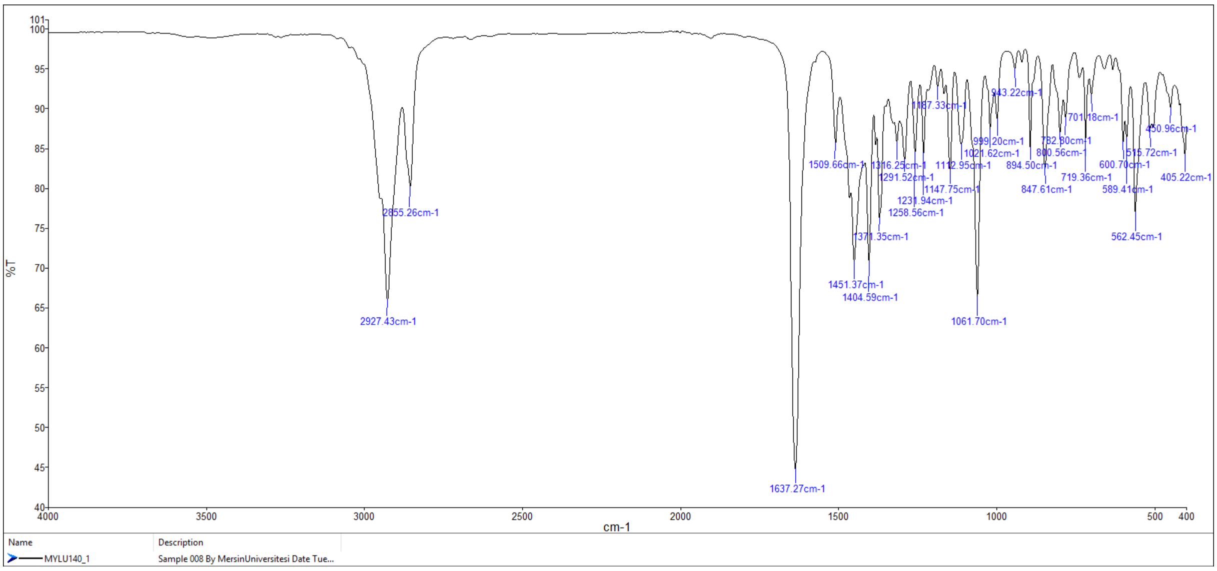

Figure S20. FTIR spectrum of (S)-N-cyclohexyl-2-(4-isobutylphenyl)-N-methylpropanamide ***(11).***

(S)-*N,N*-dibenzyl-2-(4-isobutylphenyl)propanamide (12)

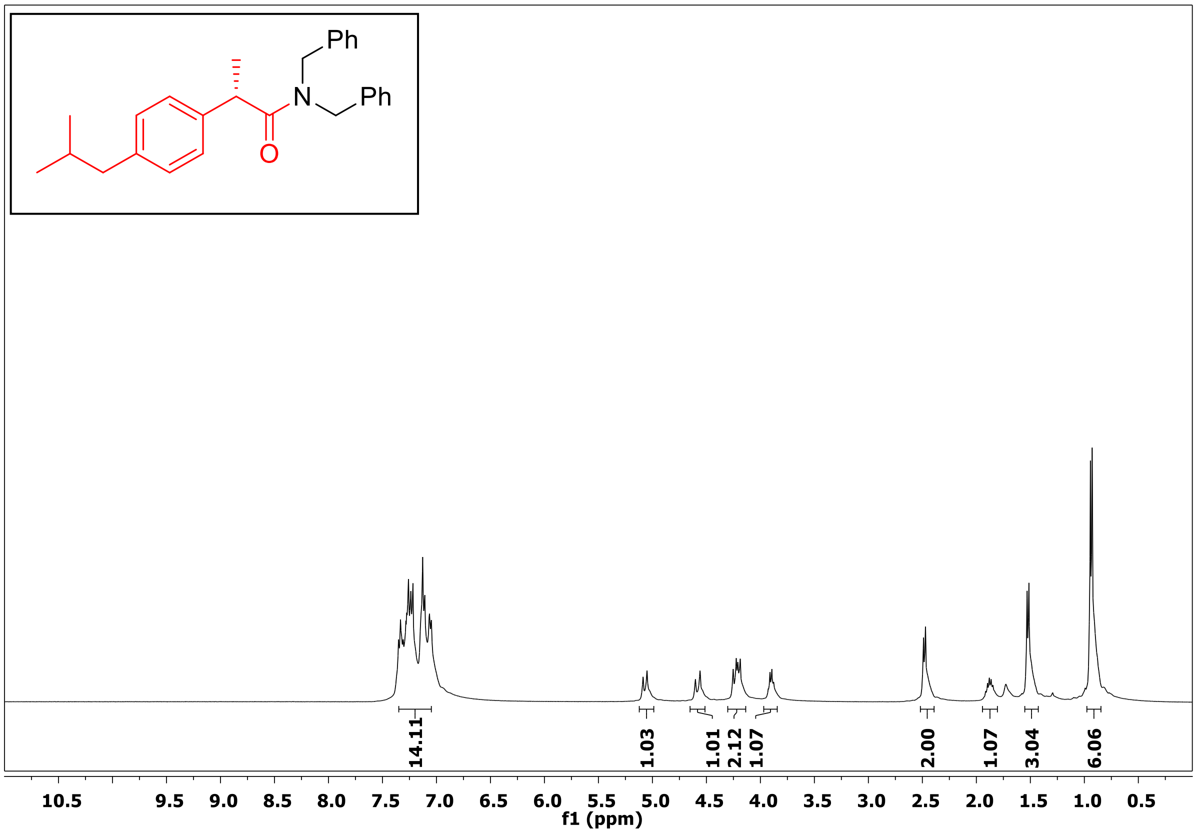

Figure S21. ^{1}H NMR spectrum of (S)-N,N-dibenzyl-2-(4-isobutylphenyl)propanamide ***(12)*** *($CDCl_3$), 400 MHz.*

(S)-*N,N*-diisobutyl-2-(4-isobutylphenyl)propanamide (13)

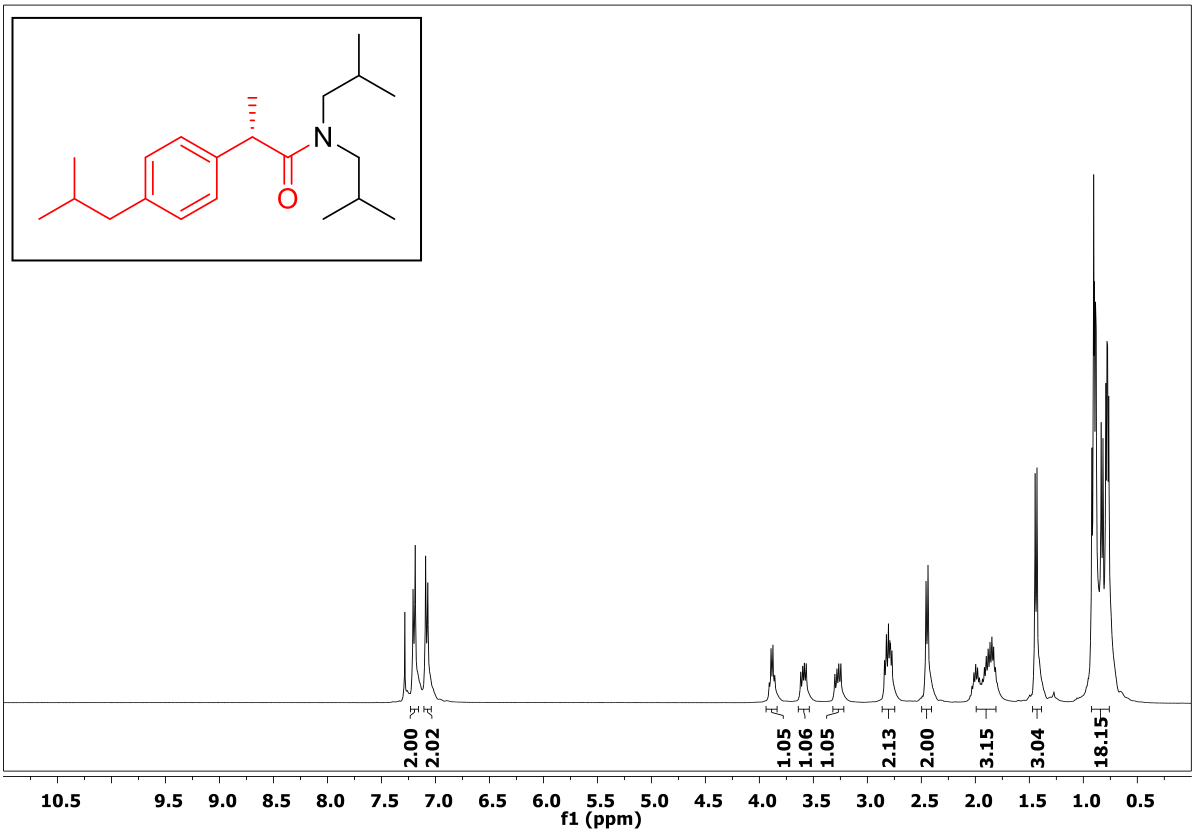

Figure S22. ^{1}H NMR spectrum of (S)-N,N-diisobutyl-2-(4-isobutylphenyl)propanamide ***(13)*** *($CDCl_3$), 400 MHz.*

(S)-*N*-benzyl-2-(4-isobutylphenyl)-*N*-((R)-1-phenylethyl)propanamide (14)

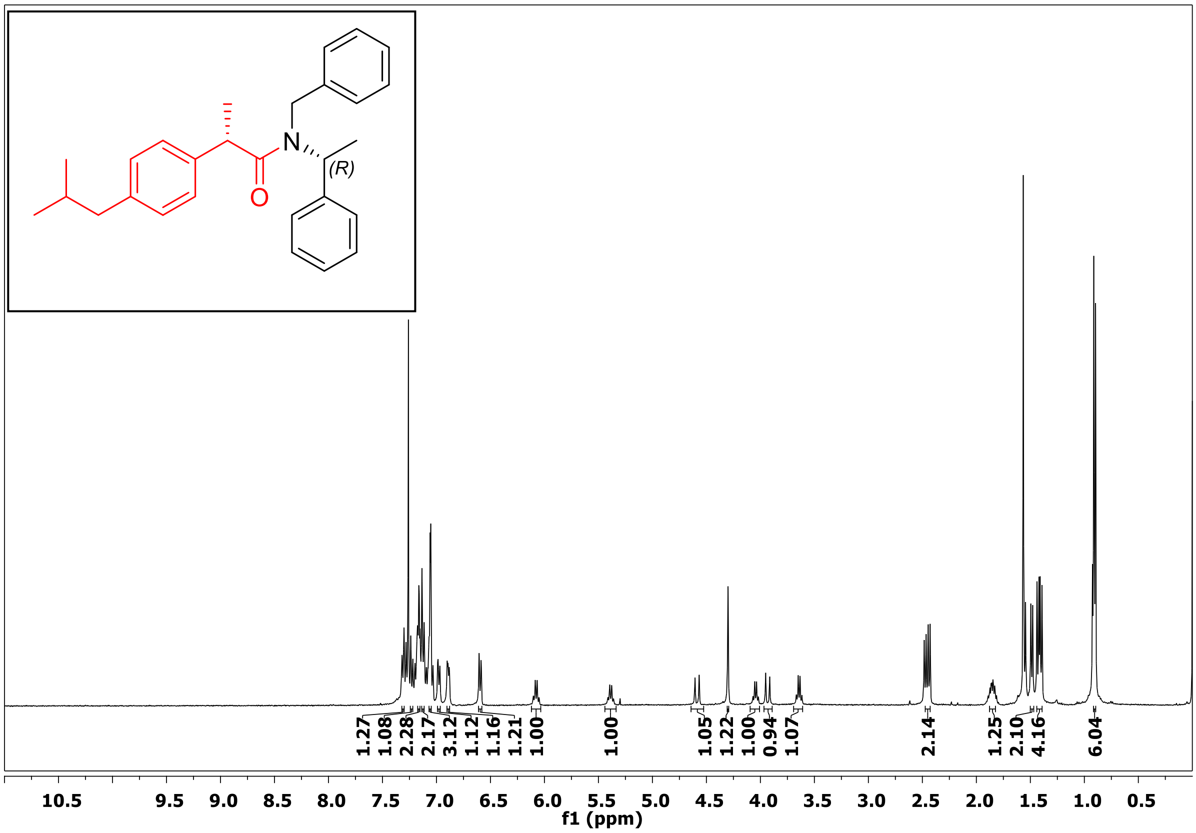

Figure S23. ^{1}H NMR spectrum of (S)-N-benzyl-2-(4-isobutylphenyl)-N-((R)-1-phenylethyl)propanamide ***(14)*** *($CDCl_3$), 400 MHz.*

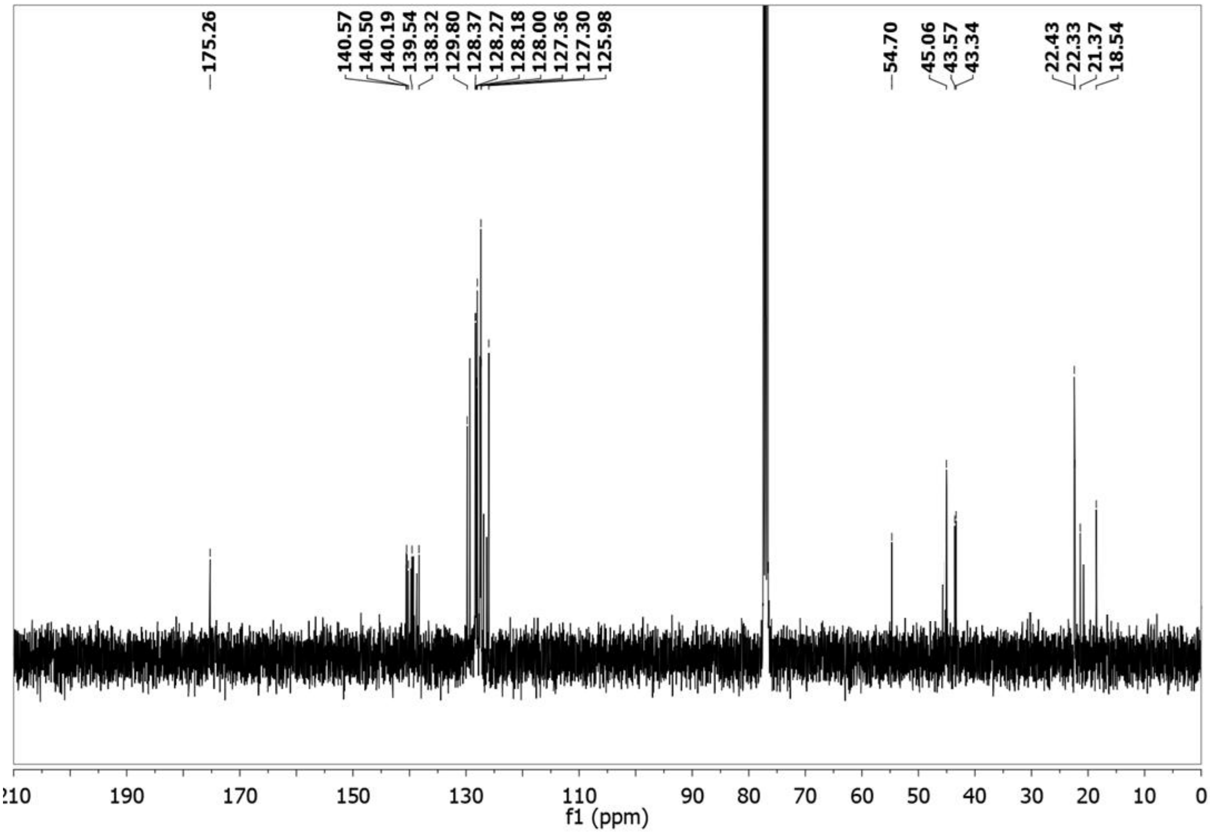

Figure S24. ^{13}C NMR spectrum of (S)-N-benzyl-2-(4-isobutylphenyl)-N-((R)-1-phenylethyl)propanamide ***(14)*** *($CDCl_3$), 101 MHz.*

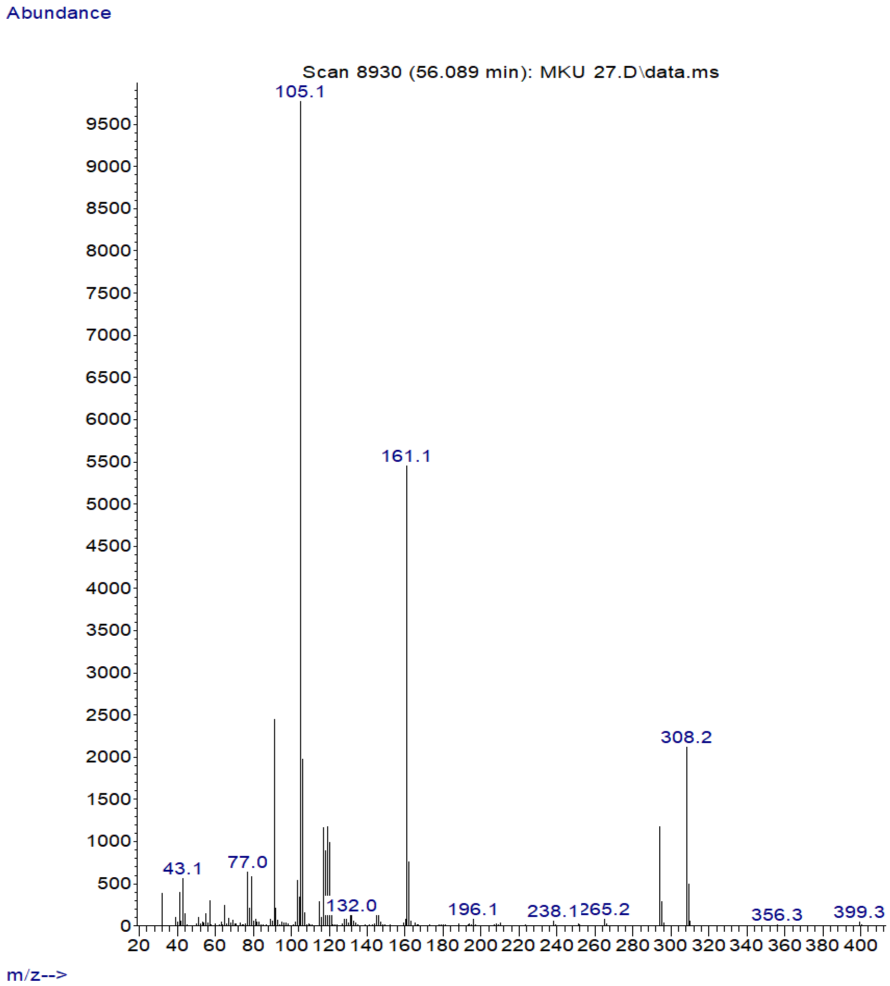

*Figure S25. MS trace of (S)-N-benzyl-2-(4-isobutylphenyl)-N-((R)-1-phenylethyl)propanamide (**14**)*

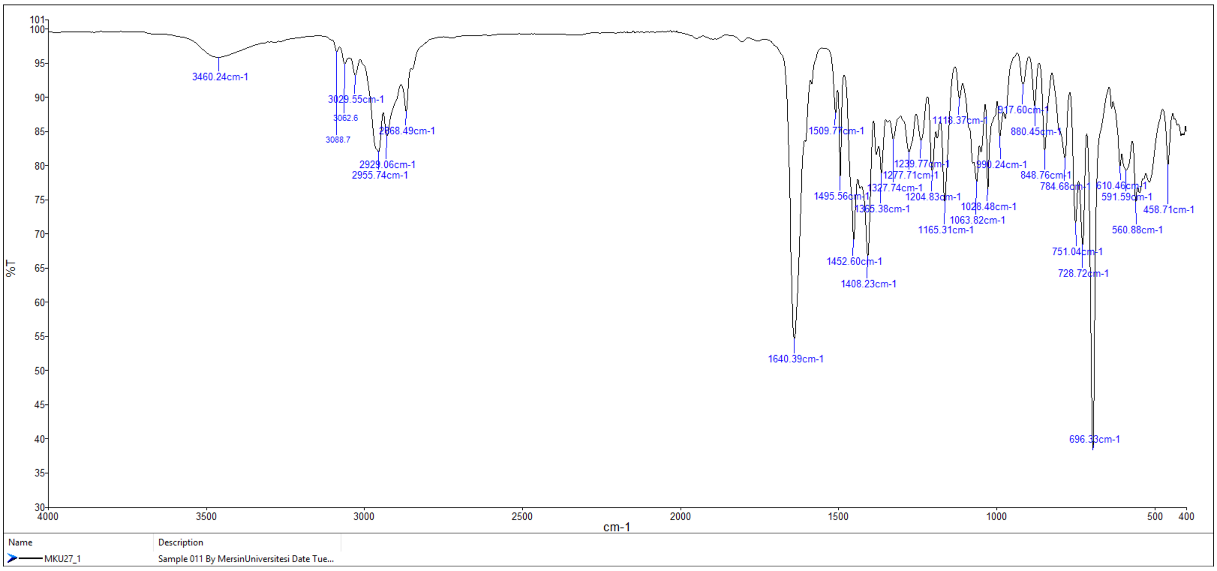

*Figure S26. FTIR spectrum of (S)-N-benzyl-2-(4-isobutylphenyl)-N-((R)-1-phenylethyl)propanamide (**14**)*

(S)-*N,N*-dicyclohexyl-2-(4-isobutylphenyl)propanamide (15)

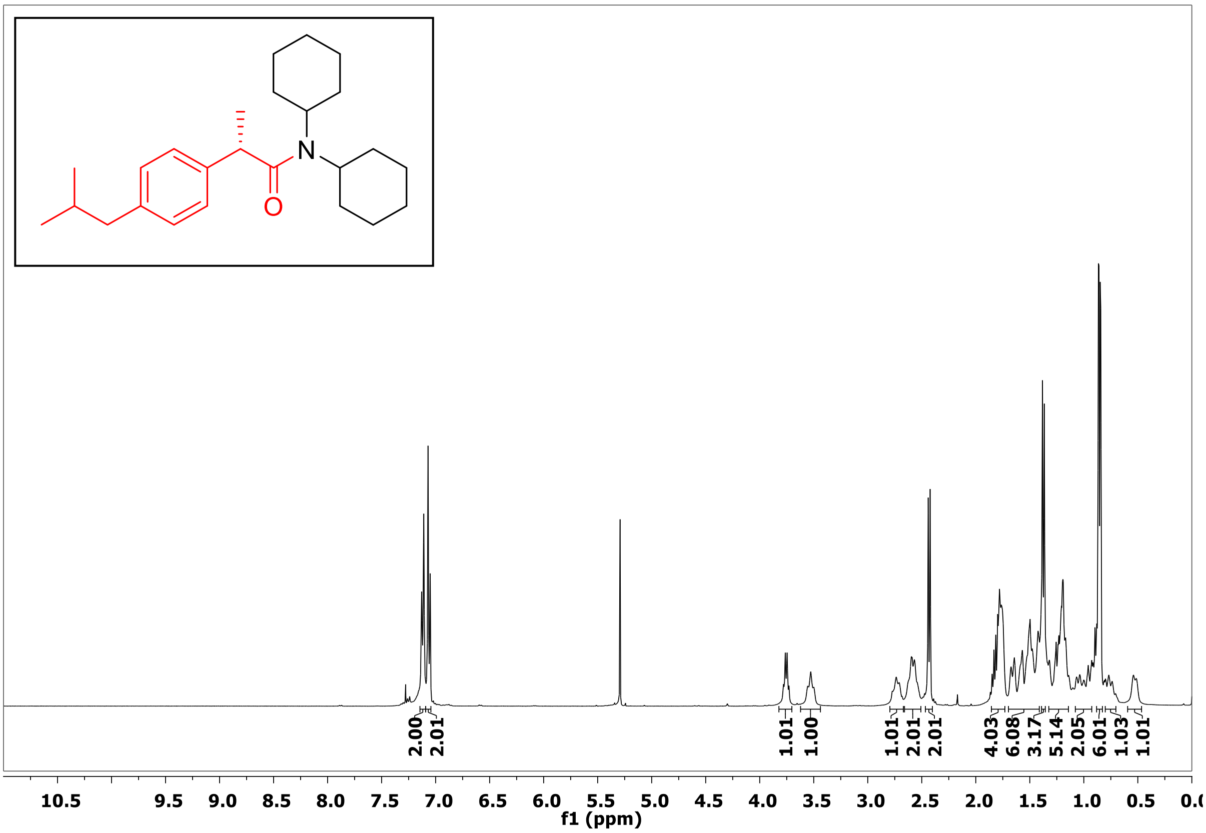

Figure S27. ^{1}H NMR spectrum of (S)-N,N-dicyclohexyl-2-(4-isobutylphenyl)propanamide ***(15)*** *($CDCl_3$), 400 MHz.*

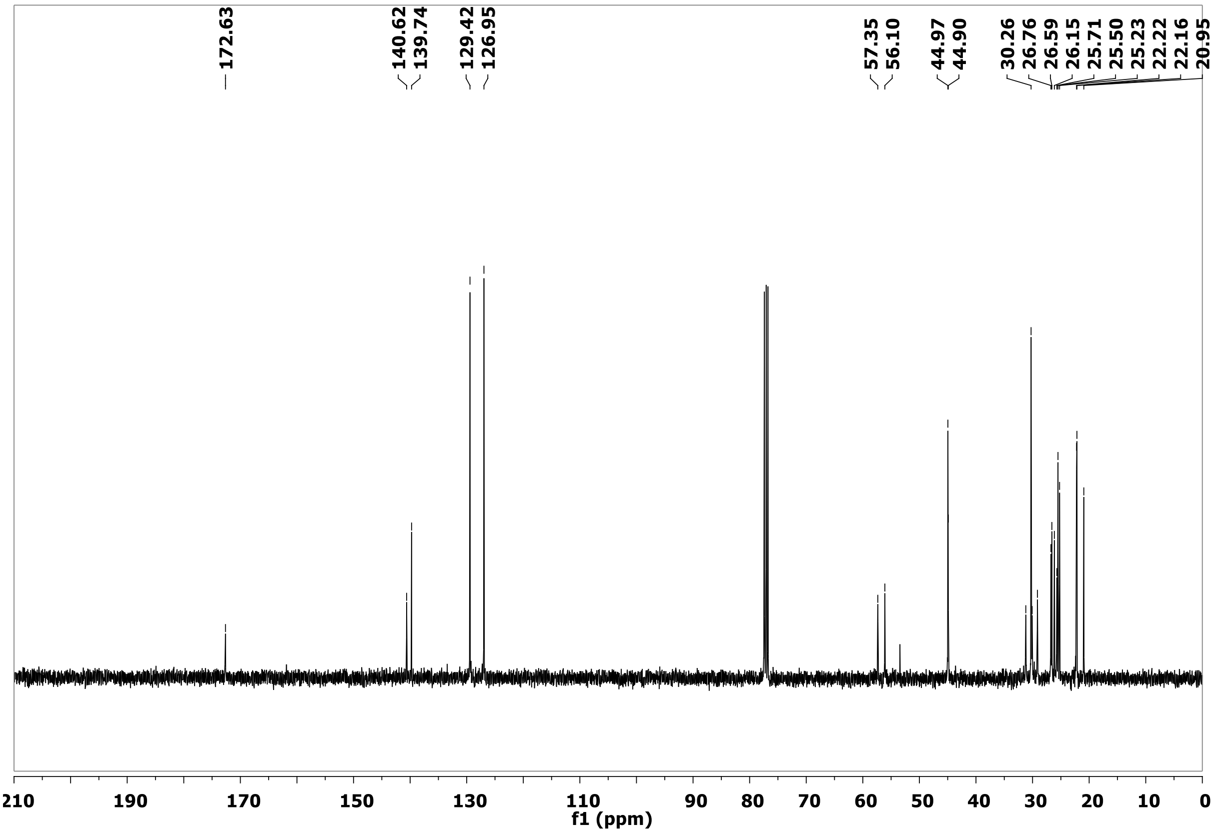

Figure S28. ^{13}C NMR spectrum of (S)-N,N-dicyclohexyl-2-(4-isobutylphenyl)propanamide ***(15)*** *($CDCl_3$), 101 MHz.*

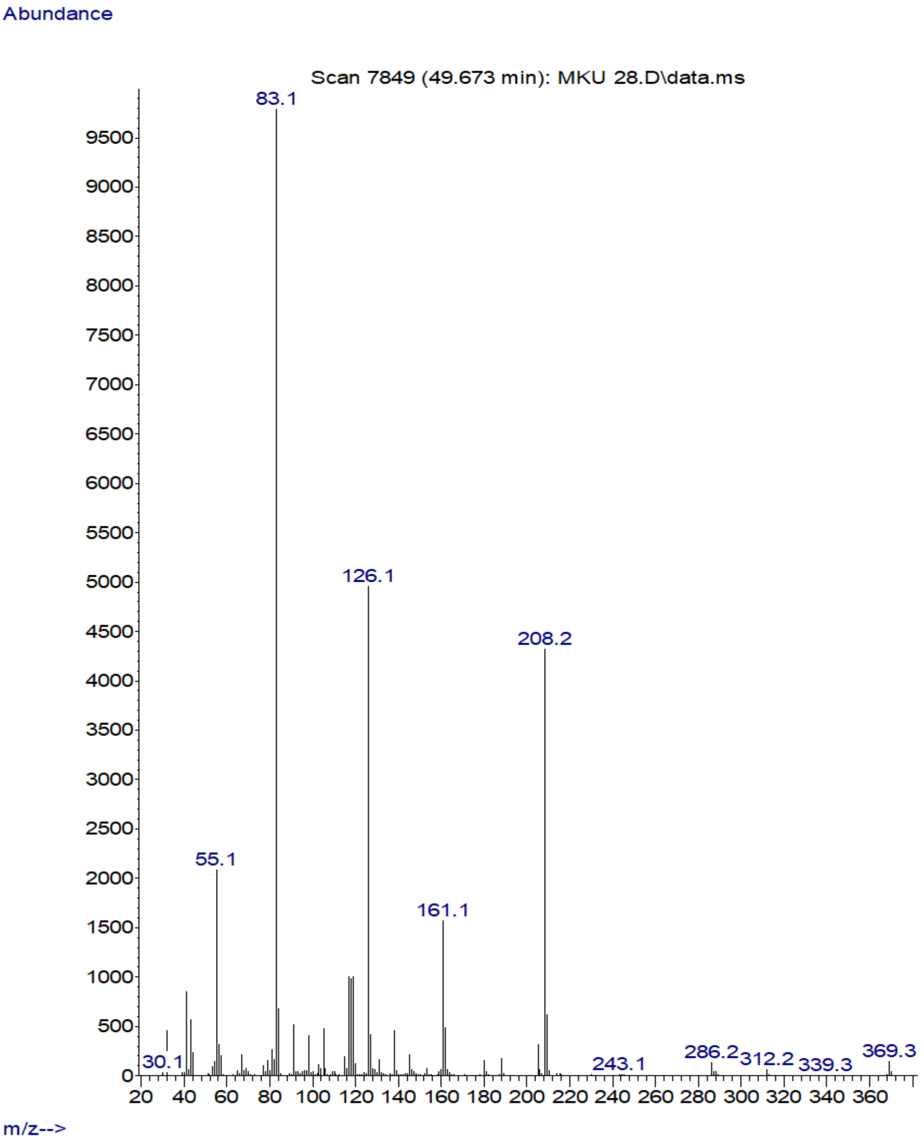

*Figure S29. MS trace of (S)-N,N-dicyclohexyl-2-(4-isobutylphenyl)propanamide (**15**)*

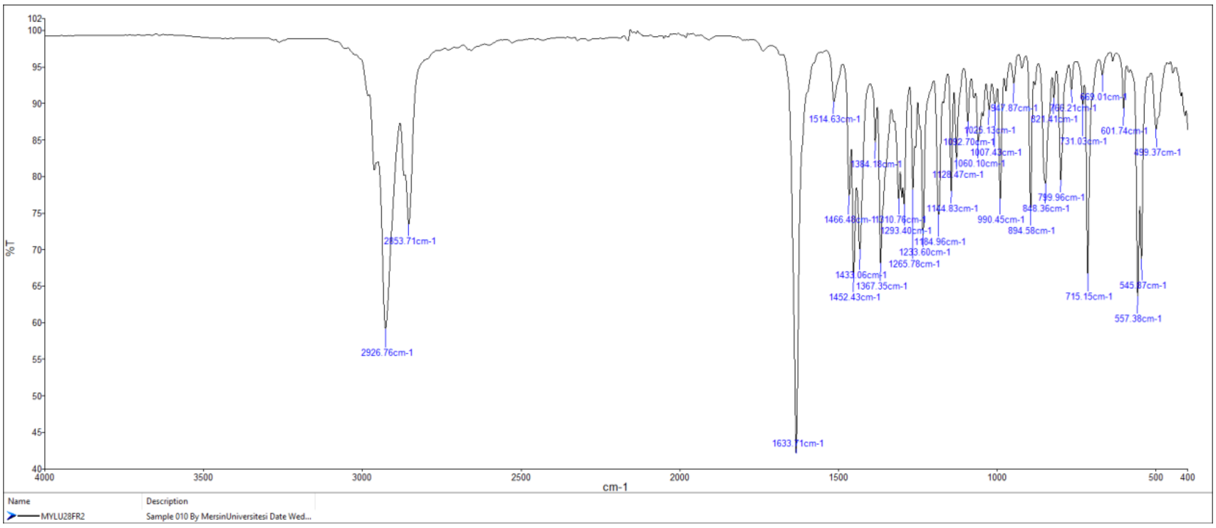

*Figure S30. FTIR spectrum of (S)-N,N-dicyclohexyl-2-(4-isobutylphenyl)propanamide (**15**)*

(S)-2-(4-isobutylphenyl)-*N,N*-bis(pyridin-2-ylmethyl)propanamide (16)

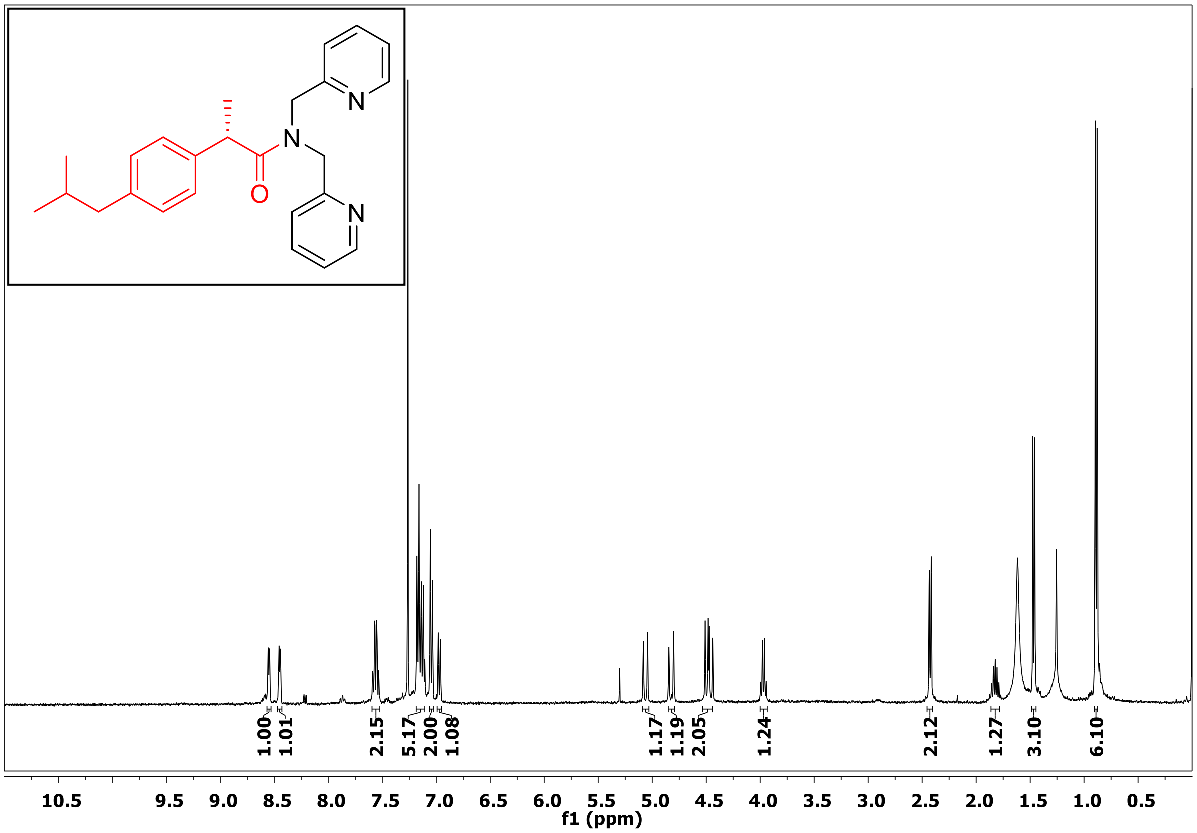

*Figure S31. ^{1}H NMR spectrum of (S)-2-(4-isobutylphenyl)-N,N-bis(pyridin-2-ylmethyl)propanamide **(16)** (CDCl$_3$), 400 MHz*

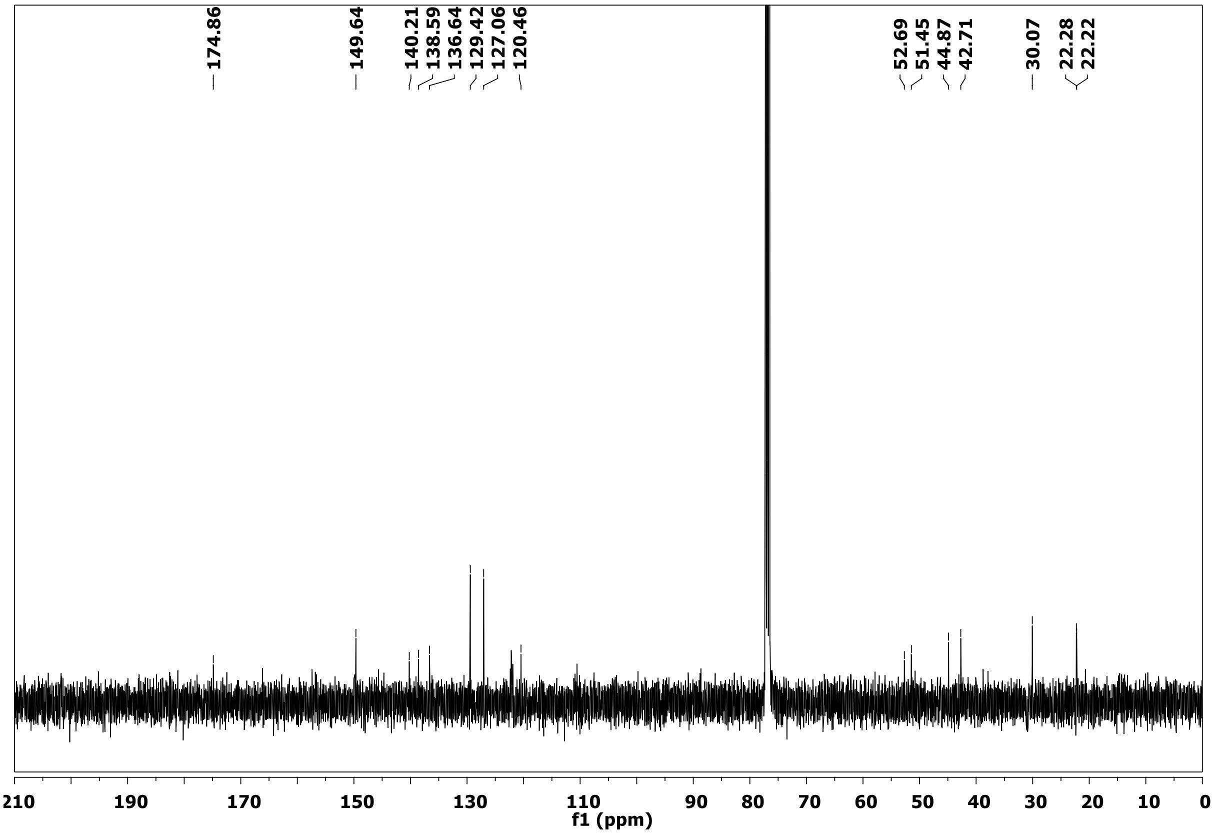

*Figure S32. ^{13}C NMR spectrum of (S)-2-(4-isobutylphenyl)-N,N-bis(pyridin-2-ylmethyl)propanamide **(16)** (CDCl$_3$), 101 MHz.*

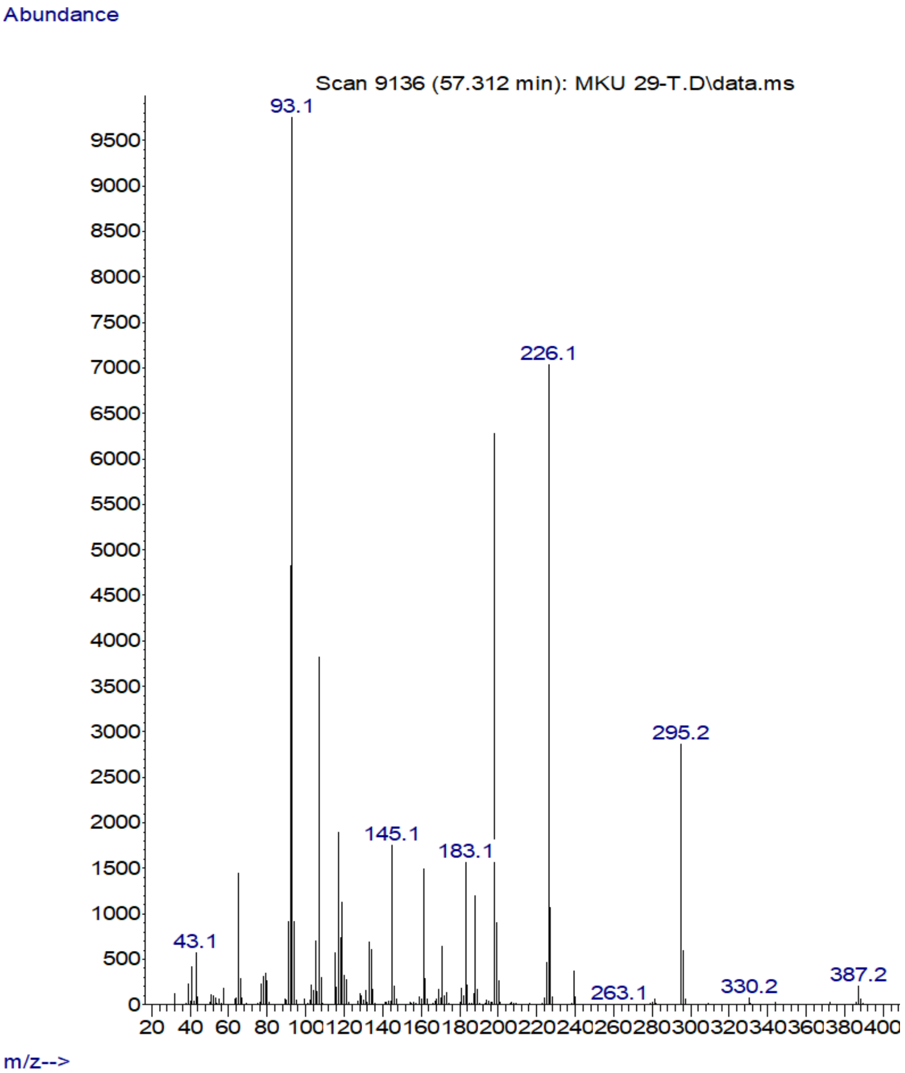

*Figure S33. MS trace of (S)-2-(4-isobutylphenyl)-N,N-bis(pyridin-2-ylmethyl)propanamide (**16**)*

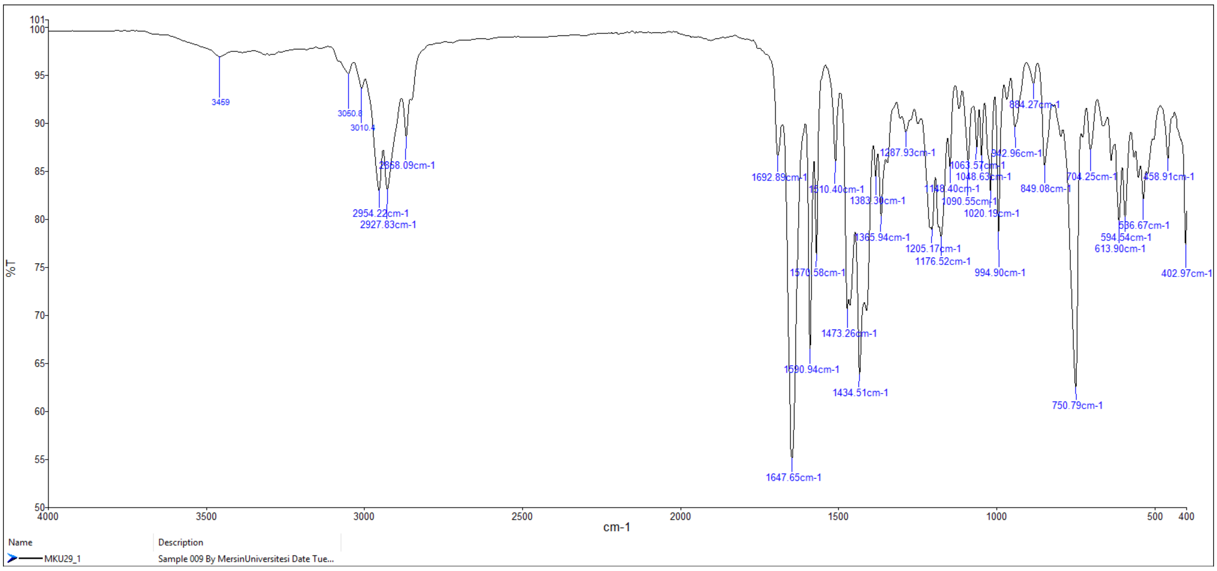

*Figure S34. FTIR spectrum of (S)-2-(4-isobutylphenyl)-N,N-bis(pyridin-2-ylmethyl)propanamide (**16**)*

ANTI-INFLAMMATORY ACTIVITIES

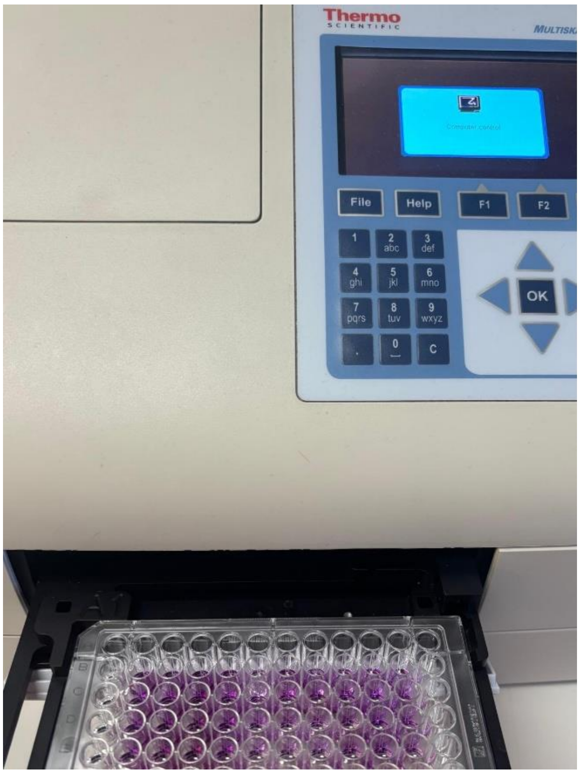

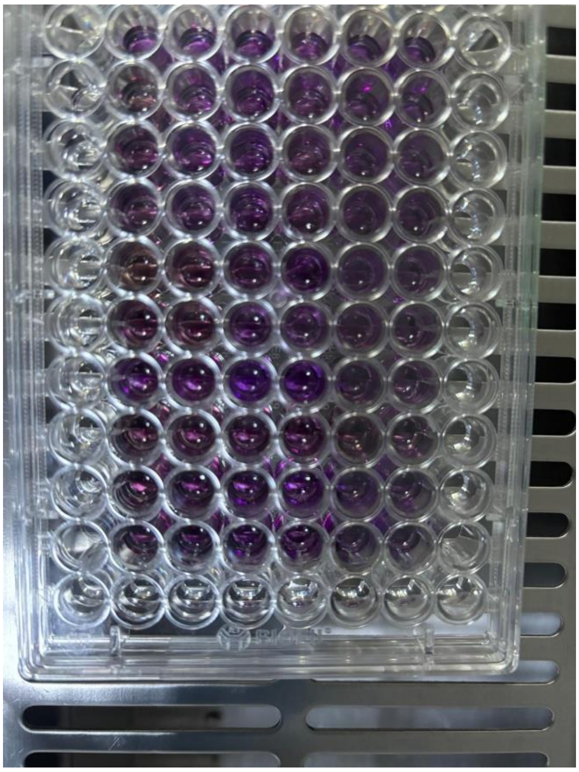

Table S1. Cytotoxicity assessment for newly prepared compounds 1 to 16 and comparison with ibuprofen via MTT assay. Cell culture in high-glucose DMEM with 10% (FBS) and 1% penicillin-streptomycin. Assessment of cell viability after 48 h of treatment with compounds or ibuprofen at specified concentrations via MTT reagent treatment, 4 h incubation, formazan dissolution with DMSO, and absorbance measurement at 570 nm. Provided values are shown as the average and the standard deviation of values obtained from four independent measurements.

Compound	**Ibuprofen**	**1**	**2**	**3**	**4**	**5**	**6**	**7**	**8**
0 (Control)	100 ±0.3	100 ±0.3	100±0.3	100 ± 0.3	100± 0.3	100 ± 0.3	100±0.3	100±0.3	100±0.3
1 µM	102±0.4	102±0.2	95±0.2	98 ± 0.1	100 ± 0.4	100 ± 0.9	100±0.5	95±5	104±0.6
25 µM	102±0.3	99±0.3	100±0.2	106 ± 0.3	115 ± 0.4	100 ± 0.2	90±0.1	86±0.1	122±0.5
100 µM	100±0.1	95±0.5	93±0.1	86 ± 0.1	96±0.2	99± 0.2	83±0.3	70±0.1	107±0.9
500 µM	92±0.4	88 ±0.6	72±0.4	78 ± 0.1	66± 0.2	77 ± 0.6	77±0.2	69±0.3	73±0.3
1000 µM	50±0.3	50±0.3	50±0.5	56 ±1	33 ± 0.1	58 ± 0.3	64±0.2	81±0.4	56±0.5
Compound	**9**	**10**	**11**	**12**	**13**	**14**	**15**	**16**	
0 (Control)	100±0.3	100 ±0.3	100 ±0.3	100 ±0.3	100 ±0.3	100 ±0.3	100 ±0.3	100 ±0.3	
1 µM	99±0.5	98±1	100±1	102±1.4	95±0.6	109±0.6	99±1	97±0.7	
25 µM	99±0.3	104±0.5	125±2	98±0.7	87±0.4	86±0.4	97±0.6	96±0.3	
100 µM	109±0.9	114±0.6	111±0.9	89±1.2	80±0.5	72±0.6	97±1.5	96±0.3	
500 µM	97±0.9	99±1	96±1	71±0.6	83± 1.2	56±0.8	83±1.4	74±0.8	
1000 µM	81±0.3	71±1	37±0.5	52±0.8	74±0.8	56±0.6	65±1	63±1	

***Table S2.** Effects of Ibuprofen and Ibuprofen Amide Derivatives on LPS-Induced Pro-Inflammatory Cytokine Production in RAW 264.7 Macrophages.

Groups	***IL-1β***	***TNF-α***	***IL-6***
Control	7.25 ± 2.38	10.10 ± 1.93	12.05 ± 0.53
LPS	69.03 ± 5.38^{a}	58.03 ± 6.02^{a}	65.73 ± 1.54^{a}
LPS + Ibuprofen	43.15 ± 1.15^{ab}	35.88 ± 3.03^{ab}	41.68 ± 2.96^{ab}
LPS + 4	14.95 ± 4.15^{bc}	15.00 ± 3.07^{bc}	9.95 ± 3.15^{bc}
LPS + 10	21.80 ± 4.70^{abc}	25.93 ± 4.64^{ab}	35.78 ± 2.42^{abd}
LPS + 11	15.95 ± 2.04^{bc}	19.23 ± 3.03^{abc}	16.75 ± 3.32^{bce}
LPS + 15	9.03 ± 2.78^{bce}	19.05 ± 0.66^{abc}	11.48 ± 3.49^{bce}
p	**< 0.001**	**< 0.001**	**< 0.001**

*Data are presented as mean ± standard deviation (SD) from four independent experiments. Statistical analysis was performed using one-way analysis of variance (ANOVA) followed by Tukey's post-hoc tests, as appropriate. Statistically significant differences at p<0.05: ^{a}compared with the Control group; ^{b}compared with the LPS group; ^{c}compared with the LPS + Ibuprofen group; ^{d}compared with the LPS + 4 group; ^{e}compared with the LPS + 10 group.

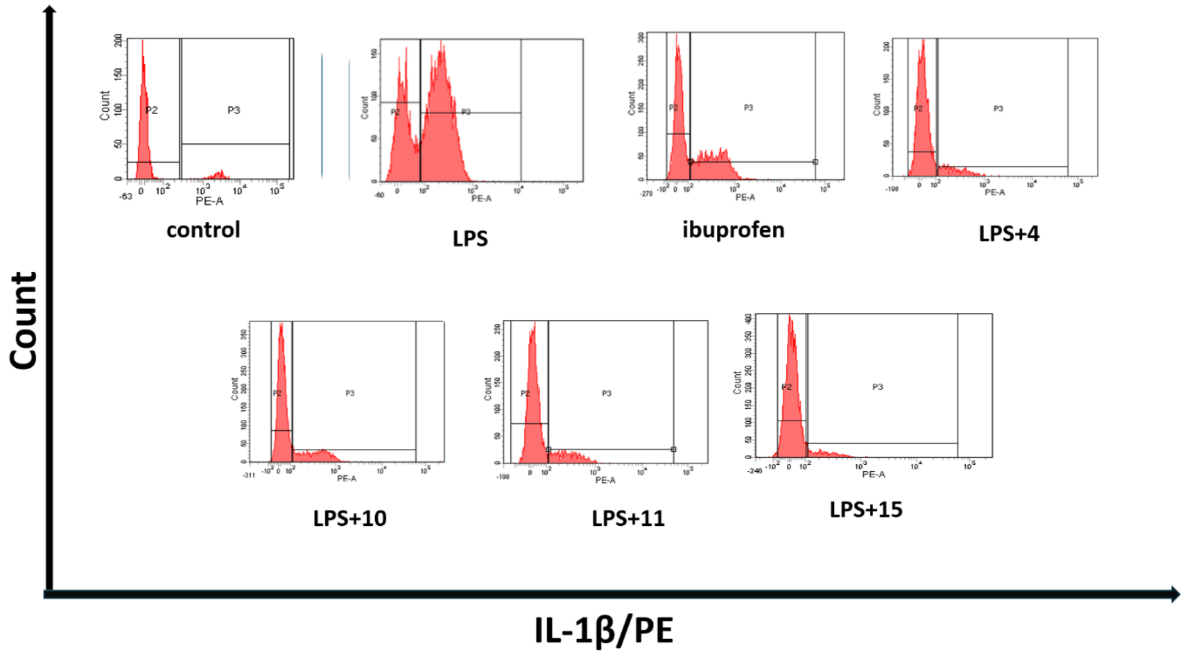

*Figure S35. Effects of ibuprofen and ibuprofen amide derivatives on LPS-induced pro-inflammatory cytokine production. RAW 264.7 macrophage cells were stimulated with lipopolysaccharide (LPS), and the effects of ibuprofen and ibuprofen amide derivatives (compounds **4**, **10**, **11**, and **15**) were evaluated. IL-1β-positive cell levels were measured in the PE channel.*

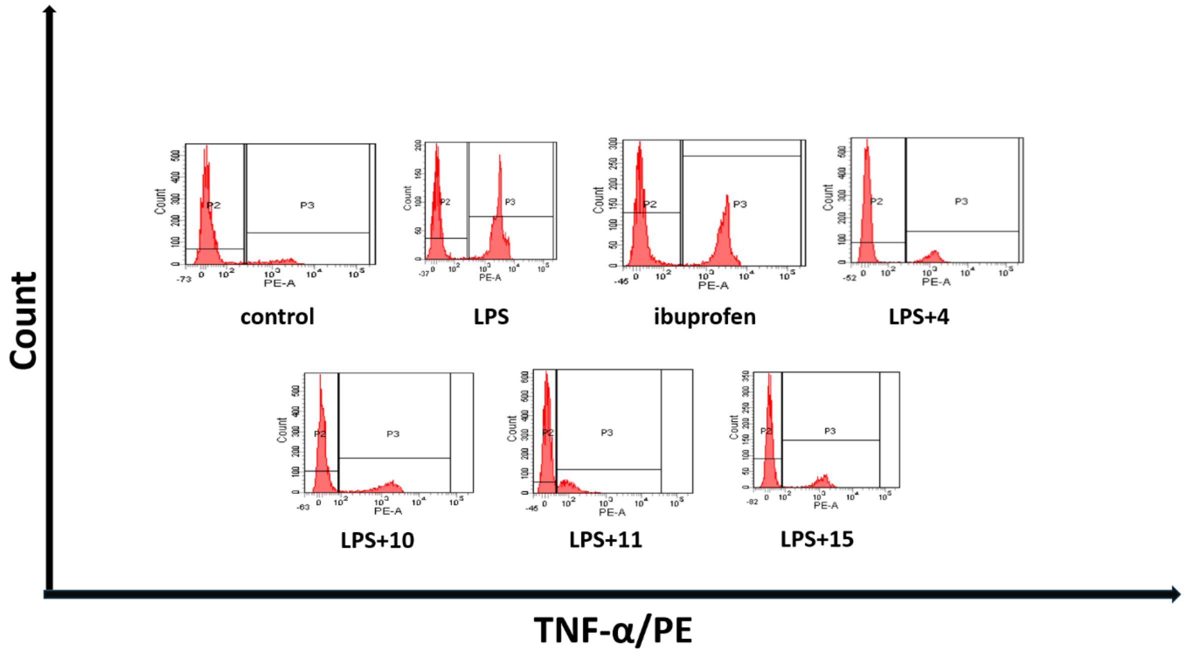

*Figure S36. The mean fluorescence intensity of TNF-α expression in activated RAW 264.7 macrophages was analyzed by flow cytometry. Cells were pretreated with ibuprofen (100 µM) for 2 hours and then stimulated with lipopolysaccharide (LPS; 1 µg/mL) for 22 hours. Additionally, cells were treated with ibuprofen and compounds **4**, **10**, **11**, and **15** for a total duration of 24 hours. Flow cytometric analyses were performed after 24 hours of treatment for all experimental groups, and the fluorescent dyes were detected in the PE channel.*

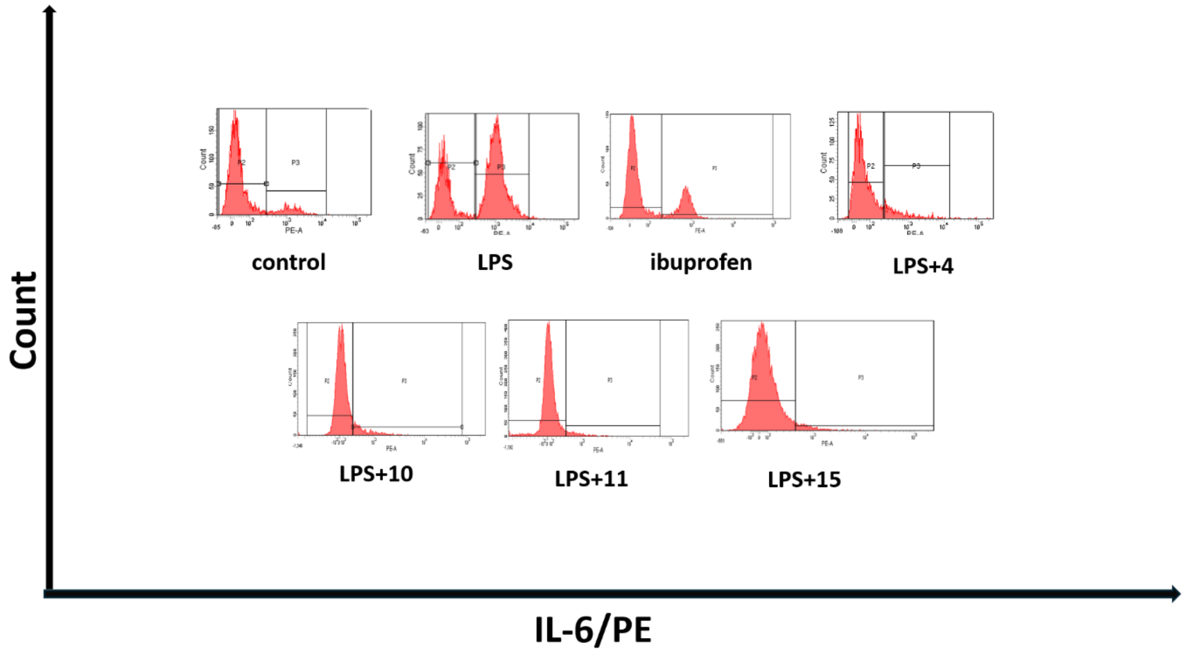

*Figure S37. The mean fluorescence intensity of IL-6 expression in activated RAW 264.7 macrophages was analyzed by flow cytometry. Cells were pretreated with ibuprofen (100 µM) for 2 hours and then stimulated with lipopolysaccharide (LPS; 1 µg/mL) for 22 hours. Additionally, cells were treated with ibuprofen and compounds **4**, **10**, **11**, and **15** for a total duration of 24 hours. Flow cytometric analyses were performed after 24 hours of treatment for all experimental groups, and the fluorescent dyes were detected in the PE channel.*

ANTI-MICROBIAL ACTIVITIES

MRSA

Table S3. In vitro anti-MRSA activity determined by OD600 assay (triplicate measurements and MIC_{99} values).

	OD-600nm				inhibition %					% inhibition average	SD		MIC99 (µg/mL)
	1	2	3		1	2	3						
**Compound8 12,5**	0,4770	0,4808	0,4772		68,2338839	68,3912958	69,06922479		**Compound8 12,5**	68,56	0,36		
**Compound8 25**	0,4704	0,4570	0,4571		68,673415	69,9559529	70,37205082		**Compound8 25**	69,67	0,72		8,67933629
**Compound8 50**	0,3893	0,3898	0,3847		74,0743207	74,3738084	75,06481722		**Compound8 50**	74,50	0,41		
**Compound8 100**	0,2911	0,2933	0,2933		80,6140117	80,7179015	80,98911071		**Compound8 100**	80,77	0,16		
**p.c.**	0,0490	0,0486	0,0485		96,7368141	96,8049438	96,85636505		**p.c.**	96,80	0,05		
**Compound15 12,5**	0,4559	0,4554	0,4540		69,6390517	70,06114	70,57298418		**Compound15 12,5**	70,09	0,38		
**Compound15 25**	0,4398	0,4390	0,4402		70,7112413	71,1393071	71,46746176		**Compound15 25**	71,11	0,31		
**Compound15 50**	0,4424	0,4400	0,4389		70,5380927	71,0735652	71,55172414		**Compound15 50**	71,05	0,41		13,7361937
**Compound15 100**	0,3358	0,3355	0,3349		77,637187	77,9435935	78,29271454		**Compound15 100**	77,96	0,27		
**p.c.**	0,0490	0,0486	0,0485		96,7368141	96,8049438	96,85636505		**p.c.**	96,80	0,05		
**Compound16 12,5**	0,1299	0,1378	0,1316		91,3492275	90,9407666	91,47005445		**Compound16 12,5**	91,25	0,23		
**Compound16 25**	0,0998	0,1058	0,1005		93,353756	93,0445073	93,48586985		**Compound16 25**	93,29	0,18		
**Compound16 50**	0,0769	0,0770	0,0770		94,878796	94,9378739	95,00907441		**Compound16 50**	94,94	0,05		6,27213021
**Compound16 100**	0,0701	0,0702	0,0709		95,3316462	95,3849188	95,40445942		**Compound16 100**	95,37	0,03		
**p.c.**	0,0490	0,0486	0,0485		96,7368141	96,8049438	96,85636505		**p.c.**	96,80	0,05		
**n.c.**	1,5016	1,5211	1,5428										

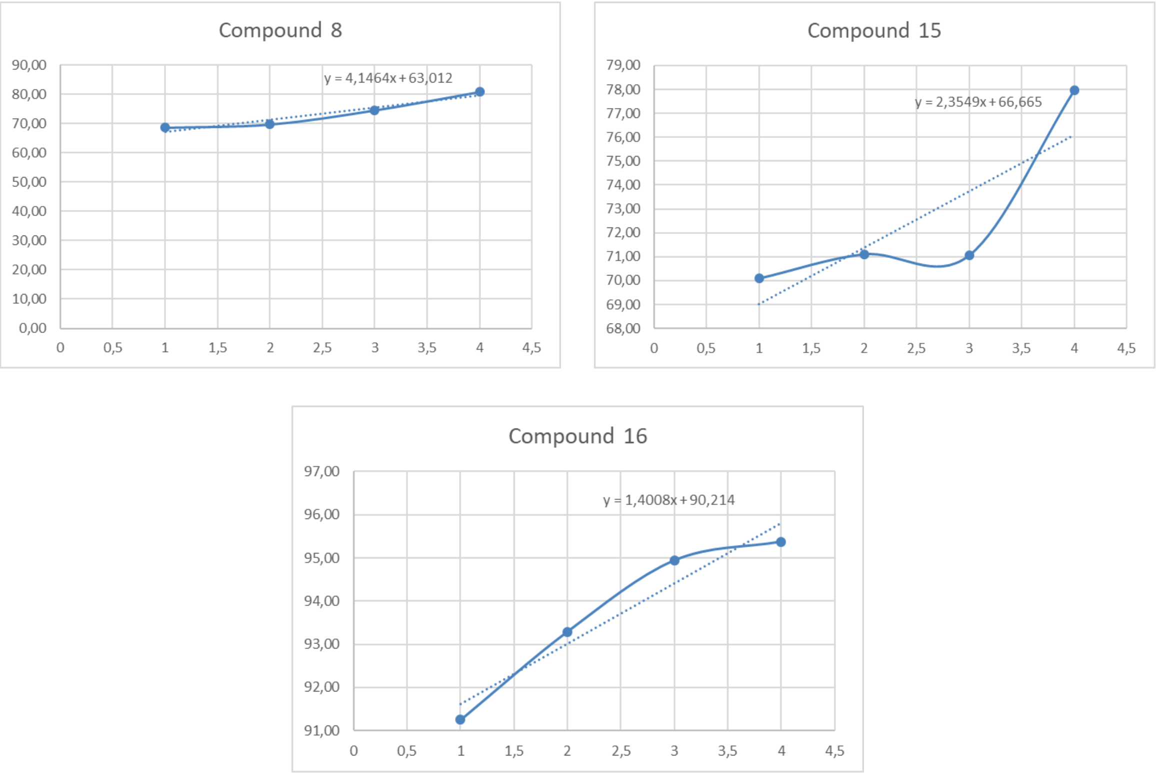

*Figure S38. Dose–response curves showing percentage growth inhibition of MRSA treated with compounds **8**, **15** and **16** (OD600 assay).*

E. COLI

Table S4. In vitro anti-*E. Coli* activity determined by OD600 assay (triplicate measurements and MIC_{99} values).

	OD-600nm				inhibition %					% inhibition average	SD	MIC99 (µg/mL)
	1	2	3		1	2	3					
**Compound15 12,5**	0,2677	0,2713	0,2705		80,970998	80,7615941	80,7910808		**Compound15 12,5**	80,8412243	0,11335024	
**Compound15 25**	0,2419	0,2460	0,2475		82,8049474	82,5556659	82,4243715		**Compound15 25**	82,5949949	0,19331213	9,25331067
**Compound15 50**	0,2029	0,2250	0,2253		85,5771965	84,0448163	84,0008522		**Compound15 50**	84,540955	0,89768064	
**Compound15 100**	0,1730	0,1751	0,1740		87,7025874	87,5833215	87,6438006		**Compound15 100**	87,6432365	0,05963496	
**Compound16 12,5**	0,2466	0,2474	0,2474		82,4708558	82,4563892	82,4314728		**Compound16 12,5**	82,4529059	0,01992124	
**Compound16 25**	0,2106	0,2110	0,2103		85,029855	85,0375833	85,0660418		**Compound16 25**	85,0444934	0,01905733	9,43279709
**Compound16 50**	0,1868	0,1867	0,1858		86,7216378	86,7607432	86,8058514		**Compound16 50**	86,7627441	0,04214248	
**Compound16 100**	0,1649	0,1657	0,1636		88,2783622	88,2498936	88,3823321		**Compound16 100**	88,3035293	0,06971384	
**p.c.**	0,0980	0,0886	0,0821									
**n.c.**	1, 4068	1,4102	1,4082									

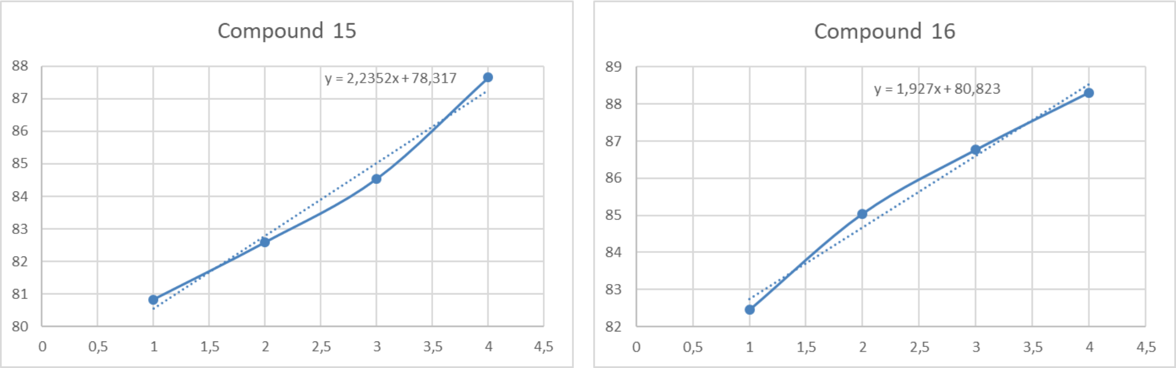

*Figure S39. Dose–response curves showing percentage growth inhibition of E.Coli treated with compounds **15** and **16** (OD600 assay).*

C. ALBICANS

Table S5. In vitro anti-C. albicans activity determined by OD600 assay (triplicate measurements and MIC_{99} values).

	OD-600nm				inhibition %					% inhibition average	SD	MIC99 (μg/mL)
	1	2	3		1	2	3					
**Compound3 12,5**	1,0100	0,9875	0,9833		20,1581028	17,7768526	22,2134325		**Compound3 12,5**	20,0494626	2,22028427	
**Compound3 25**	0,9236	0,9194	0,9167		26,9881423	23,4471274	27,482003		**Compound3 25**	25,9724242	2,20086749	13,4869211
**Compound3 50**	0,9071	0,9022	0,9039		28,2924901	24,8792673	28,4945811		**Compound3 50**	27,2221128	2,03147833	
**Compound3 100**	0,7309	0,7356	0,7327		42,2213439	38,7510408	42,0378135		**Compound3 100**	41,0033994	1,95275708	
**Compound15 12,5**	0,7076	0,7115	0,7035		44,0632411	40,7577019	44,3477573		**Compound15 12,5**	43,0562334	1,99566348	
**Compound15 25**	0,5725	0,5793	0,5791		54,743083	51,7651957	54,1887509		**Compound15 25**	53,5656765	1,58370436	6,75504831
**Compound15 50**	0,5021	0,5023	0,4977		60,3083004	58,1765196	60,6281149		**Compound15 50**	59,7043116	1,33273468	
**Compound15 100**	0,3361	0,3325	0,3174		73,43083	72,3147377	74,891227		**Compound15 100**	73,5455982	1,29207314	
**Compound16 12,5**	0,9144	0,9168	0,9135		27,715415	23,6636137	27,7351475		**Compound16 12,5**	26,3713921	2,34502565	
**Compound16 25**	0,7337	0,7176	0,7136		42	40,2497918	43,5487699		**Compound16 25**	41,9328539	1,6505137	9,19444839
**Compound16 50**	0,6371	0,6349	0,6305		49,6363636	47,1357202	50,1226169		**Compound16 50**	48,9649003	1,60266503	
**Compound16 100**	0,5928	0,5930	0,5953		53,1383399	50,6244796	52,9072067		**Compound16 100**	52,2233421	1,38946988	
**p.c.**	1,2650	1,2673	1,2641									
**n.c.**	0,1009	0,1201	0,0913									

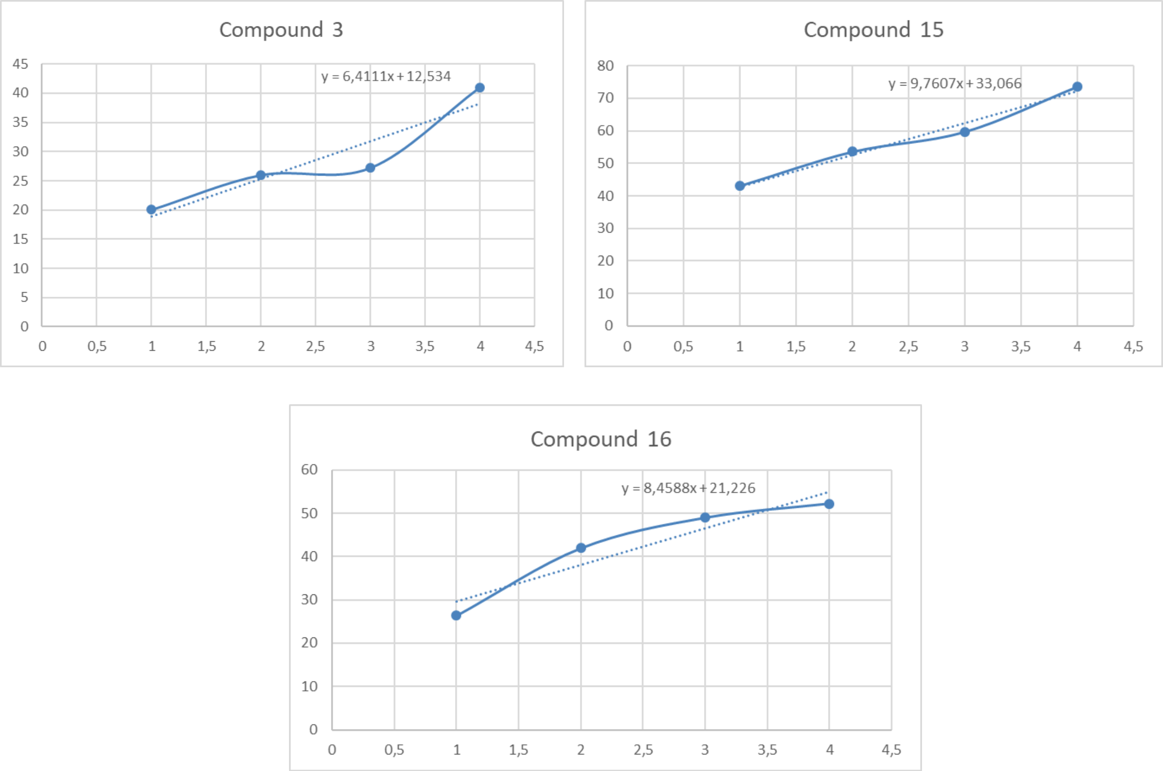

*Figure S40. Dose–response curves showing percentage growth inhibition of C.albicans treated with compounds **3**, **15** and **16** (OD600 assay).*

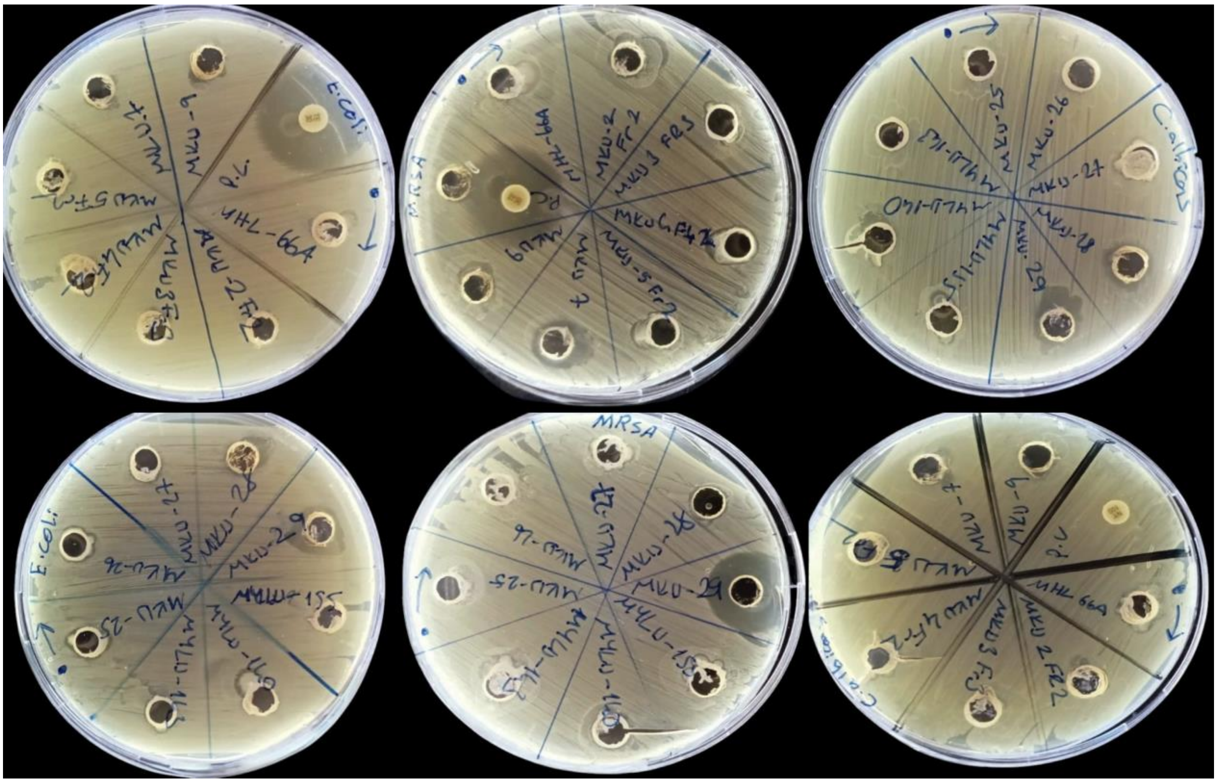

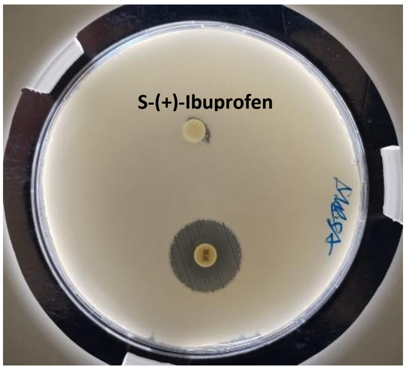

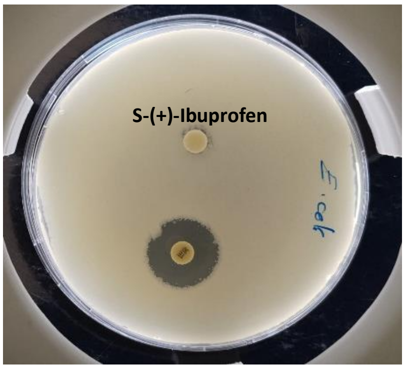

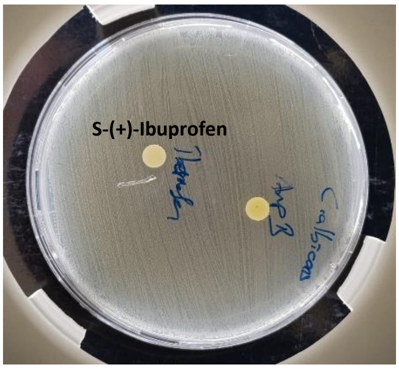

Table S6. Stock solution

Name	Concentration (mg/ml)	Name	Concentration (mg/ml)
Compound 1	32,1	Compound 9	17,6
Compound 2	30,1	Compound 10	20,8
Compound 3	18	Compound 11	30,1
Compound 4	32,3	Compound 12	30,5
Compound 5	26,7	Compound 13	32,6
Compound 6	27,6	Compound 14	35,7
Compound 7	12,8	Compound 15	14,9
Compound 8	22,7	Compound 16	3,4
